# Flower abscission in *Vitis vinifera* L. triggered by gibberellic acid and shade discloses differences in the underlying metabolic pathways

**DOI:** 10.3389/fpls.2015.00457

**Published:** 2015-06-22

**Authors:** Sara Domingos, Pietro Scafidi, Vania Cardoso, Antonio E. Leitao, Rosario Di Lorenzo, Cristina M. Oliveira, Luis F. Goulao

**Affiliations:** ^1^Linking Landscape, Environment, Agriculture and Food, Instituto Superior de Agronomia, Universidade de LisboaLisbon, Portugal; ^2^Agri4Safe-BioTrop, Instituto de Investigação Científica Tropical I.P., LisbonPortugal; ^3^Dipartimento di Scienze Agrarie e Forestali, University of PalermoPalermo, Italy

**Keywords:** abscission, gibberellic acid, grapevine, inflorescence, metabolomics, shade, thinning

## Abstract

Understanding abscission is both a biological and an agronomic challenge. Flower abscission induced independently by shade and gibberellic acid (GAc) sprays was monitored in grapevine (*Vitis vinifera* L.) growing under a soilless greenhouse system during two seasonal growing conditions, in an early and late production cycle. Physiological and metabolic changes triggered by each of the two distinct stimuli were determined. Environmental conditions exerted a significant effect on fruit set as showed by the higher natural drop rate recorded in the late production cycle with respect to the early cycle. Shade and GAc treatments increased the percentage of flower drop compared to the control, and at a similar degree, during the late production cycle. The reduction of leaf gas exchanges under shade conditions was not observed in GAc treated vines. The metabolic profile assessed in samples collected during the late cycle differently affected primary and secondary metabolisms and showed that most of the treatment-resulting variations occurred in opposite trends in inflorescences unbalanced in either hormonal or energy deficit abscission-inducing signals. Particularly concerning carbohydrates metabolism, sucrose, glucose, tricarboxylic acid metabolites and intermediates of the raffinose family oligosaccharides pathway were lower in shaded and higher in GAc samples. Altered oxidative stress remediation mechanisms and indolacetic acid (IAA) concentration were identified as abscission signatures common to both stimuli. According to the global analysis performed, we report that grape flower abscission mechanisms triggered by GAc application and C-starvation are not based on the same metabolic pathways.

## Introduction

Abscission is the process by which vegetative and reproductive organs can be detached from plants as an effect of developmental, hormonal, and environmental cues. Abscission control is regarded as an important agricultural concern because it affects production yield and quality. In fruit species, fruit set is often excessive and the natural drop, which enables the plant to self-regulate its load, is not sufficient to satisfy fresh market quality standards (Bonghi et al., 2000). Fruit thinning is a common practice, particularly in table grape production, in which the reduction of berry number per bunch is mandatory to guarantee improved bunch appearance and quality and decreased diseases incidence (Dokoozlian and Peacock, 2001). Excluding labor-demanding manual thinning, the most common thinning method for table grapes uses chemical treatments with GAc sprays at bloom to induce flower abscission. The effectiveness of this practice is known to vary due to both internal (such as cultivar, phenologic stage, physiological condition, and age) and external (such as nutrient availability, irrigation, temperature, irradiation, and humidity) conditions (Weaver et al., 1962; Hopping, 1976; Looney and Wood, 1977; Dokoozlian, 1998; Dokoozlian and Peacock, 2001; Reynolds and Savigny, 2004; Reynolds et al., 2006; Hed et al., 2011). Gibberellins participate in biological processes such as cell elongation, dormancy breaking, parthenocarpy induction, and seed germination (Yamaguchi, 2008; Davière and Achard, 2013). However, despite the widespread use of GAc spraying, the mechanisms by which GAc works as thinning agent are not fully understood. According to the photosynthates competition hypothesis (Dokoozlian, 1998), GAc stimulates general organ growing, inducing competition for nutrients between flowers and shoots and/or among flowers within the inflorescence, which leads to reductions in the amount of nutrients available for berry set and growth. Alternatively, GAc can be responsible for unbalancing hormone relative concentrations, in agreement with the hormone balance hypothesis (Dokoozlian, 1998). Auxins are known to regulate gibberellin endogenous levels (Yamaguchi, 2008). A flow of inhibitory auxin in an organ destined to abscise prevents cell separation until its endogenous levels drop, de-repressing ethylene, which then activates the transcription of CW disassembly related genes (Else et al., 2004; Dal Cin et al., 2005). The effect of shade imposition to promote berry set reduction has been first investigated by Roubelakis and Kliewer (1976) and Ferree et al. (2001). The use of this practice as an alternative thinning method was successful also in other species, such as apple (Schneider, 1975; Byers et al., 1985, 1990, 1991; Corelli Grappadelli et al., 1990; Widmer et al., 2008; Zibordi et al., 2009; Basak, 2011) and involves intercepted light reduction during a short period of time after bloom. The pronounced reduction of net photosynthetic rate under shading promotes the competition for photoassimilates between vegetative and reproductive organs, leading to shedding of the later, which have less sink strength at this early stage of development (Vasconcelos et al., 2009). Hence, abscission stands as challenging biological question since it can be induced by agents that apparently act by promoting opposite changes to the plant physiology. However, although the hormone and the assimilate theories may look contrasting, changes in assimilate availability may be the trigger required for changing hormone balances, leading to abscission. Moreover, sugars are more than an energy source as may also act as messengers operating in gene expression regulation or as signaling molecules (Lebon et al., 2008).

The mechanisms underlying organ abscission, were recently reviewed by Estornell et al. (2013), and involve signal peptides and specific receptors, mostly regulated by hormones, in which ethylene, ABA, and jasmonic acid act as accelerating signals. Conversely, auxin, gibberellins, PAs, and brassinosteroids act in abscission inhibiting signaling. The developmental phenomenon of physiological drop represents the natural reduction of fruit set and enables the plant to shed the weaker sinks, regulating the fruit load according to its capacity to produce the metabolic energy required to complete the development of reproductive and vegetative structures (Bonghi et al., 2000). Natural drop occurs after an increased ABA and ethylene production that induces a negative feedback in fruit development, as demonstrated in apple (Botton et al., 2011). In wine grapes, natural drop was showed to be related to lower sugar and PAs availability for developing flowers (Aziz, 2003; Lebon et al., 2004). Declines in the sugar supply at meiosis results in excessive flower abortion in this species (Lebon et al., 2008) which together with the expression of sucrose- or hexose-transporter genes (Davies et al., 1999), suggests a role for sugars in flower stress avoidance. Free-PA synthesis is also closely related to the onset of ovarian development and retards abscission (Aziz, 2003). Since PAs and ethylene share SAM as a common intermediate, SAM may be alternatively channeled toward the PA pathway, functioning as an alternative control. Free PAs fluctuate in parallel with sugars in the grape inflorescence, suggesting also a contribution in the modulation of their concentrations (Aziz, 2003).

Changes on the biochemical and transcriptome profiles during flower and fruit abscission triggered by growth regulators (Whitelaw et al., 2002; Dal Cin et al., 2005, 2009; Li and Yuan, 2008; Meir et al., 2010; Botton et al., 2011; Giulia et al., 2013; Peng et al., 2013; Wang et al., 2013) or by low light conditions (Aziz, 2003; Zhou et al., 2008; Li et al., 2013) have been studied in several species such as tomato (*Solanum lycopersicon*), apple (*Malus domestica*), lychee (*Litchi chinensis*), or wine grapes aiming at understanding the effect caused by chemical/environmental perturbations. The above-cited studies revealed a coordinated response of hormones, ROS, sugar metabolism, and signaling pathways to determine the downstream activation of abscission which includes increased activity of CW-modifying enzymes. Nonetheless, to our knowledge, only one publication to date (Zhu et al., 2011) reports a direct comparison of the mechanisms underlying abscission triggered by two distinct cues. The authors compared napththaleneacetic acid (NAA) and shading treatments in inducing abscission in apple, through transcriptome analysis, and observed shared pathways involving reduction of photosynthesis, carbon transport and signaling, and hormone crosstalk. The aim of the present study was to provide a first global approach for understanding the changes occurring in vine inflorescences and canopy that explain flower abscission in *Vitis vinifera* L. (Black Magic table grape cultivar), triggered by two contrasting abscission-inducing treatments (shade and GAc spraying) under conditions that leading to similar berry shed rates. The goal was to search for specificities and common links in metabolic pathways that control abscission.

## Materials and Methods

### Experimental Conditions and Design

**FIGURE 1:**
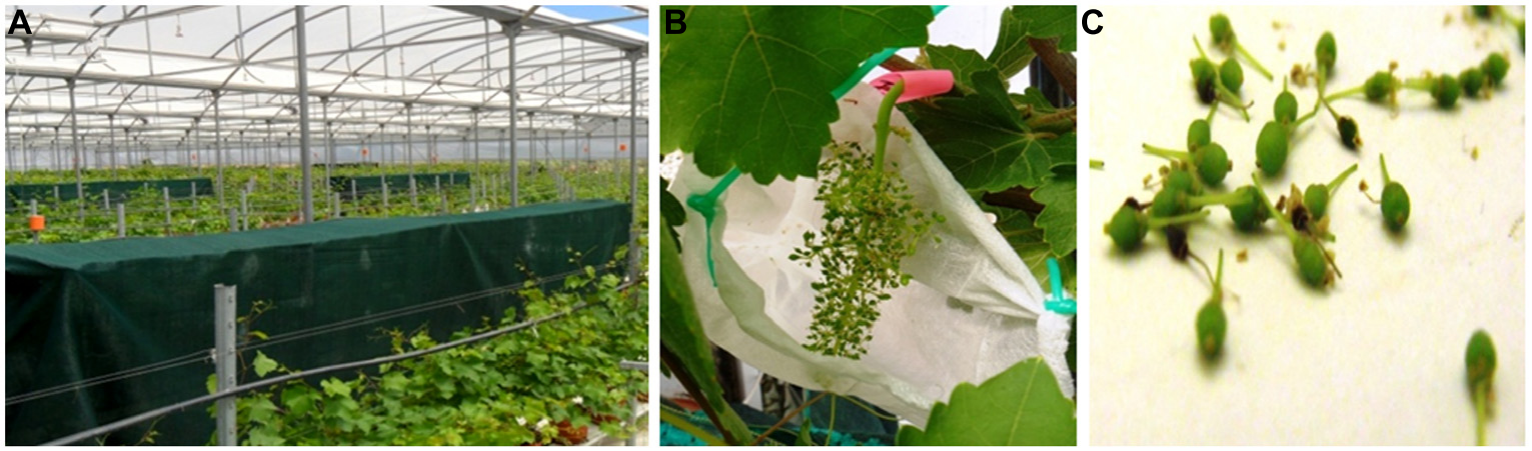
**Aspect of the experimental table grape vines (Black Magic cv) growing in greenhouse conditions **(A)**, monitoring of flowers drop **(B)** and flowers detached from the base of flower pedicel **(C)** after abscission-inducing treatments application**.

The trails were carried out in a greenhouse in the south of Sicily, in a soilless table grape commercial production system (Di Lorenzo et al., 2014) of ‘Black Magic’ vines (*V. vinifera* L.) own-rooted in 2010 (**Figure 1A**). Black Magic is a very early seeded table grape cultivar, with high fertility and yield, released by the National Research Institute of Grape and Wine in Chisinau, Moldova. The assays were performed during the late production cycle of 2011 and the early production cycle of 2012. Plants were spaced 1.60 m between lines × 0.40 m between plants, trained as unilateral cordon pruned with six buds and managed following integrated fertilization, irrigation, and pest-management practices (Di Lorenzo et al., 2009). The number of fertirrigations ranged between 5 and 20, judged by monitoring microclimate conditions. Nutritive solutions had the composition of 3, 1.25, 0.5, 0.65, 0.75, 0.5, 1.25, 7, 0.75, 2, and 0.5 mM of Ca, Mg, Na, K, NH_4_, Si, P, NO_3_, HCO_3_/CO_3_, SO_4_, and Cl, respectively, the pH was 5.0 and the electrical conductivity was maintained between 1.5 and 2 ms cm^-1^. Treatments were: (i) reduction of intercepted light and (ii) chemical thinning with GAc, both established at bloom (50% cap fall, stage 65 of the BBCH scale Lorenz et al., 1994). Shade treatments entailed covering the vines with green polypropylene 90% nets (Serroplast, Italy) for a period of 12 days. Chemical treatment consisted in spraying GAc (Gibberellin 1.8% GA_3_, Gobbi, Italy) at 15 ppm concentration. A group that remained untreated was included as control. Experiments were designed in three randomized blocks by treatment with 14 vines each, using single vines as replicates. Climate conditions were monitored above the canopy of shaded and control vines (WatchDog MicroStation, Spectrum Tech., USA; Supplementary Figure S1).

#### Late Production Cycle

Plants were kept stored cold until June 2011 and the experiments started at 3rd July. The 50% cap fall stage (bloom) occurred after 34 days and harvest was carried out 67 DAB. This production cycle lasted a total of 101 days. The day (7 a.m. to 7 p.m.)/night (7 p.m. to 7 a.m.) mean temperatures registered were 32/23°C, relative humidity was 41/64% and PAR was 5/504 μmol m^-2^ s^-1^.

#### Early Production Cycle

The experiments were conducted in 2012 using the same plants as in the previous year. The early production cycle started at 9 February, 50% cap fall stage occurred after 53 days and grapes were harvested 77 DAB. This cycle lasted a total of 130 days. The recorded day/night mean temperatures were 26/14°C, relative humidity was 45/79% and PAR was 17/566 μmol m^-2^ s^-1^.

### Vine Physiology Monitoring

Flower and berry drop were monitored by positioning non-woven cloth bags around bunches at 50% cap fall after the imposition of each treatment, to collect the shed flowers (**Figures 1B,C**). Flowers were collected and counted, 2, 4, and 12 DAB in 10 bunches per treatment. Bunches were selected taking uniformity of bloom in account. At harvest, the same bunches were collected and the final number of berries was recorded to calculate the cumulative and daily rate berry drop percentages. Net Pn, E and *gs* were measured in the morning period (9:00–11:00 a.m.) using a portable infrared gas analyzer (CIRAS, PPsystems, UK) on 12 mature leaves from the central part of the shoot, twice during the shade period (at 3 and 10 DAB). Shoot length, estimated leaf chlorophyll content (SPAD-502 m, Minolta, Japan) and total (sum of primary and secondary) leaf area (WinDIAS leaf area measurement system, Delta-T Devices, UK) were determined 12 DAB, before removal of the shade nets, in nine shoots per treatment.

### Metabolic Analysis

#### Quantification of Target Metabolites

Sugar (glucose, sucrose, fructose, and stachyose), free PA (putrescine, spermine, spermidine, and cadaverine) and hormone (IAA and ABA) contents extracted from inflorescence samples collected in the late cycle, 1, 3, and 4 DAB were quantified by high performance liquid chromatography (HPLC), aiming at determining the metabolic changes explaining flower abscission. The biochemical analyses were conducted using liquid nitrogen frozen powdered samples of whole inflorescences deprived from the rachis. Samples for soluble sugars quantification (100 mg) were extracted according to Damesin and Lelarge (2003), and samples were injected into a HPLC (Beckman Coulter, USA) and separated on a Sugar-Pak I column I (300 × 6.5 mm, Waters) at 90°C under a 122 μM EDTA-Ca solution and a flow rate of 0.5 ml min^-1^. Peaks were detected by RI (Refractive Index Detector 2414, Waters). Free PAs were quantified according to Smith and Davies (1987) with modifications. Samples (100 mg) were mixed with 300 μL of a 5% perchloric acid solution, kept for 50 min in ice and centrifuged for 20 min at 20000 *g* at 4°C. Saturated Na_2_CO_3_ (200 μl) and dansyl chloride (400 μL, 5 mg ml^-1^ in acetone) were added to 100 μl of the supernatant, and mixtures were incubated in the dark at 60°C for 1 h. Proline (10 mg) was then added and further incubated for 30 min. PAs were extracted with 500 μl of toluene, the organic phase was dried under nitrogen and the residue was dissolved in 300 μl acetonitrile. The resulting samples were injected into the HPLC (Ultimate 3000, Dionex, Sunnyville, CA, USA), eluted through a C18 column (particle size 5 μm, 4.6 × 150 mm, Thermo Scientific) at a flow rate of 1 ml min^-1^ with a mobile phase consisting of 10% acetonitrile solution, pH 3.5 (solvent A) and acetonitrile (solvent B) using a 60 to 90% of solvent A gradient, during 23 min. Peaks were detected with a diode array detector (DAD) at 346 nm. IAA and ABA were extracted according to Kelen et al. (2004) with modifications. Samples (200 mg) were extracted with 600 μL of 70% methanol and incubated at 4°C overnight. The extraction was repeated twice and the methanol evaporated under vacuum. 0.1 M phosphate buffer (800 μl) was added to the aqueous phase and partitioned with 300 μl of ethyl acetate three times. After ethyl acetate removal, the pH was adjusted to 2.5 with 1 N HCl. The solution was further partitioned three times with 450 μl of diethyl ether, passed through anhydrous sodium sulfate, evaporated at 50°C under vacuum and the residue was dissolved in 100 μl of methanol. Aliquots were injected into the HPLC (Ultimate 3000, Dionex, Sunnyville, CA, USA), eluted through a C18 column (particle size 5 μm, 4.6 × 150 mm, Thermo Scientific) under a 30 mM phosphoric acid solution with 26% acetonitrile at 4 pH during 30 min at 0.8 ml min^-1^ and the peaks were detected with a DAD at 208 and 265 nm. In all cases, extractions were done in duplicate readings, each from three biological replicates per treatment. Standards for peak identification were purchased from Sigma-Aldrich^®^.

#### Global Metabolomic Profile

Sample points for metabolomic analysis were chosen based on the significant changes observed after target chromatography quantifications. Therefore, samples from three biological replicates (200 mg) of GAc-, shaded-treated and control inflorescences collected at 4 DAB in the late production cycle, were lyophilized, methanol extracted, and analyzed using the integrated platform developed by Metabolon^®^ (Durham, USA) consisting of a combination of three independent approaches: (1) ultrahigh performance liquid chromatography/tandem mass spectrometry (UHLC/MS/MS2) optimized for basic species, (2) UHLC/MS/MS2 optimized for acidic species, and (3) gas chromatography/mass spectrometry (GC/MS). Methods were followed as previously described (Evans et al., 2009; Ohta et al., 2009).

### Evaluation of Productivity and Berry Quality Attributes

The final number of shot berries (parthenocarpic small berries that remain green at harvest) and regular-sized berries, bunch weight, rachis length and weight, bunch compactness (ratio between total number of berries and length of the rachis) and yield per plant (product of the bunch weight by number of bunches per plant) were recorded and calculated at harvest in the same 10 bunches per treatment used for flower drop monitoring. Ten berries per bunch were randomly selected to measure berries weight and diameter. The remaining berries were distributed in three samples per treatment to measure total soluble solids (TSS; in °Brix using a PR-32 refractometer, Atago, Japan) and titratable acidity (TA; by potentiometric titration with 0.1 N NaOH up to pH 8.1).

### Data Imputation and Statistical Analysis

To access the significance of the differences observed between treatments and production cycles, variance analysis (one- and two-way ANOVA) and *post hoc* (Tukey’s HSD with α = 0.05) tests were conducted using Statistix 9 (Analytical Software, Tallahassee, FL, USA). To improve adjustment to the normal distribution, percentage values were arcsin sqrt(x) transformed and values concerning number of berries were square-root transformed. For global metabolomic analyses, raw area counts for each biochemical were rescaled by dividing each sample’s value by the median value for the specific metabolite. Following log_2_ transformations, statistical analysis of the data was performed using Array Studio (Omicsoft). In order to visualize the results, a heat map was generated to show fold change (FC) defined as the log_2_ of the means ratio of each treatment and control for each compound (Supplementary Figure S2). Welch’s two-sample *t*-tests were used to determine whether each metabolite had significantly increased or decreased in abundance. False discovery rates (FDRs) were calculated as *q*-values according to Storey and Tibshirani (2003) to account for the large number of tests. Metabolites that significantly changed in response to at least one of the imposed treatments were used to conduct correlation matrix-based principal component analysis (PCA) and hierarchical clustering. Dendrograms associated with the heatmap and approximately unbiased and bootstrap probability *P*-values were computed using pvclust version 1.3.2 (Suzuki and Shimodaira, 2006) with the UPGMA method and 1000 bootstrap replications. Box plots were generated for those compounds that showed a significant increase or decrease using both the Welch two-sample *t*-test, FDR (i.e., *P* < 0.05 and *q* < 0.10) significance values and |FC| ≥ 1. Mapping of named metabolites was performed onto general biochemical pathways, provided in the Kyoto Encyclopedia of Genes and Genomes (KEGG^1^) and Plant Metabolic Network (PMN^2^).

## Results

### Effect of GAc and Shade on Flower Abscission

**FIGURE 2:**
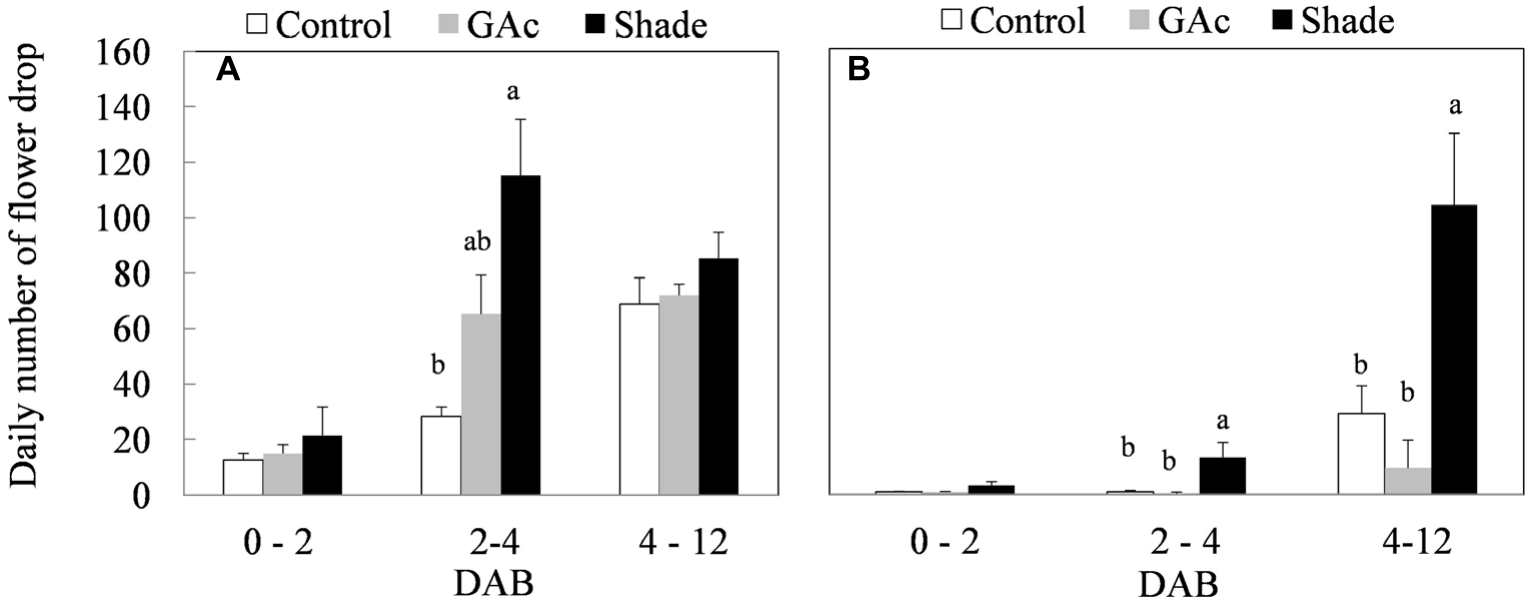
**Average daily number of flower drop in summer **(A)** and spring **(B)** production cycles as effect of GAc and shade treatments on ‘Black Magic’ vines (mean ± SE).** Within each sampling date, different letters indicate statistically significant differences (*P* < 0.05).

The purpose of the treatments was to induce flower abscission, triggered by two distinct stimuli, with distinct physiological basis. In the late production cycle, both shade and GAc treatments resulted in higher cumulative percentages of berry drop (95.9% in the shade and 94.3% in the GAc treatment) comparing to the natural drop values observed in control bunches (81.0%; **Table 1**). Similarly, the average daily number of berries drop was highest in the shade treatment (115 ± 20 berries dropped per bunch per day), followed by GAc (62 ± 14 berries dropped per bunch per day) and lower in the control (28 ± 4 berries dropped per bunch per day) between 2 and 4 DAB (**Figure 2A**). In the early production cycle, shade imposition was the treatment that promoted the highest percentage of berries drop (49.4%; **Table 1**). This effect was reflected by an average higher daily number of dropped berries during 2–4 and 4–12 DAB intervals (13 ± 5 and 104 ± 26 berries dropped per bunch per day, respectively), when compared to control (1 ± 0.5 and 29 ± 10 berries dropped per bunch per day, respectively) and GAc treatments (0.3 ± 0.2 and 10 ± 3 berries dropped per bunch per day, respectively; **Figure 2B**). Based on these results, the metabolic composition of samples collected in the late cycle, treated with hormonal and light stress abscission-inducing signals, was analyzed.

**Table 1:** Effect of shade and GAc treatments on the average percentage of flower drop, total leaf area, and estimated leaf chlorophyll content at 12 DAB, on net photosynthetic rate (Pn) and *gs* during the shade period in ‘Black Magic’ vines in late and early cycles.

Production cycle	Treatment	Cumulative flower drop (%)	Leaf area (m^2^ vine^-1^)	Shoot length (cm)	Leaf chlorophyll content (spad units)	Pn (μmol m^-2^ s^-1^)	*gs* (mol m^-2^ s^-1^)
Late	Control	81.0 b	0.52	79.4	30.5	2.72 a	227.57
	GAc	94.3 a	0.62	96.3	30.3	2.12 a	251.81
	Shade	95.9 a	0.58	86.8	31.7	0.26 b	153.4
Early	Control	16.9 b	1.86	174.6	28.7 ab	3.23 a	576.96 a
	GAc	5.4 b	1.83	183.4	28.1 b	3.18 a	613.69 a
	Shade	49.4 a	1.92	158.7	31.2 a	0.04 b	268.89 b

Within each column, different letters indicate significant differences (*P* < 0.05) among treatments, independently in each production cycle, according to Tukey’s HSD test.

### Impacts on Vine Physiology

Natural flower drop was significantly affected by environmental factors, exerting a significant effect on fruit set (**Table 1**). A higher drop rate occurred in the late production cycle (81%) when compared to the early cycle (16.9%). Comparing shaded with unshaded conditions, a 90% PAR reduction was observed, while no significant differences in temperature and relative humidity were perceived (Supplementary Figure S1). On clear sunny day conditions, the 90%-interception shade cloth provided approximately a maximum PAR of 157 and 170 μmol m^-2^ s^-1^ in late and early cycles, respectively, which demonstrates the strong net Pn reduction achieved under shaded conditions, in the magnitudes of 90 and 99%, in the late and early cycle, respectively. Transpiration rate (E; not shown) and *gs* decreased under shade, only during the early production cycle, by 23 and 54%, respectively, when compared to controls (**Table 1**). No differences in shoot length and total leaf area were observed between treatments. Nevertheless, in the early cycle, a higher estimated leaf chlorophyll content was perceived in shaded plants (31.2 spad units) when compared with plants treated with GAc (28.1 spad units; **Table 1**). Production cycles and the interaction production cycles × treatment were statistically significantly different regarding cumulative flower drop and *gs* (*P* < 0.01). Production cycle also affected leaf area, shoot length and leaf chlorophyll content (*P* < 0.01; **Table 1**), impacting final bunch morphology and berry quality (**Table 2**).

**Table 2:** Effect of shade and GAc treatments on bunch and berries characteristics at harvest in Black Magic table grape cultivar in the late and early cycles.

Production cycle	Treatment	Yield (kg plant^-1^)	Bunch weight (g)	No berries	No shot berries	Rachis length (cm)	Rachis weight (g)
Late	Control	1.9 a	315.9 a	96.8 a	22.3	15.0 ab	7.7 b
	GAc	1.1 b	193.2 b	62.1 b	29.1	17.4 a	10.4 a
	Shade	0.9 b	148.3 b	46.2 b	14.3	12.0 b	4.2 c
Early	Control	8.9 a	879.8 a	173.0 a	188.3 b	24.1 a	12.9 a
	GAc	5.6 b	555.0 b	105.5 b	407.1 a	23.2 a	10.5 ab
	Shade	5.7 b	562.3 b	93.4 b	117.6 b	20.2 b	7.8 b

		**Bunch compactness**	**Berry diameter (cm)**	**Berry weight (g)**	**TSS (°Brix)**	**TA (g L^-1^)**

Late	Control	8.0 a	14.1 a	3.83 a	12.5 b	5.7
	GAc	6.1 ab	13.2 c	3.47 b	14.1 ab	5.1
	Shade	5.1 b	13.7 b	3.36 b	15.5 a	5.4
Early	Control	15.5 b	17.2 ab	5.15 c	13.9	3.8
	GAc	22.1 a	16.6 b	5.18 b	14.3	4.7
	Shade	10.5 c	17.8 a	5.78 a	15.8	3.8

Values represent the average of the appropriate number of replicates. Within each column, different letters indicate significant differences (*P* < 0.05) among treatments individually in each production cycle according to Tukey’s HSD test.

### Impacts on Metabolite Content

**FIGURE 3:**
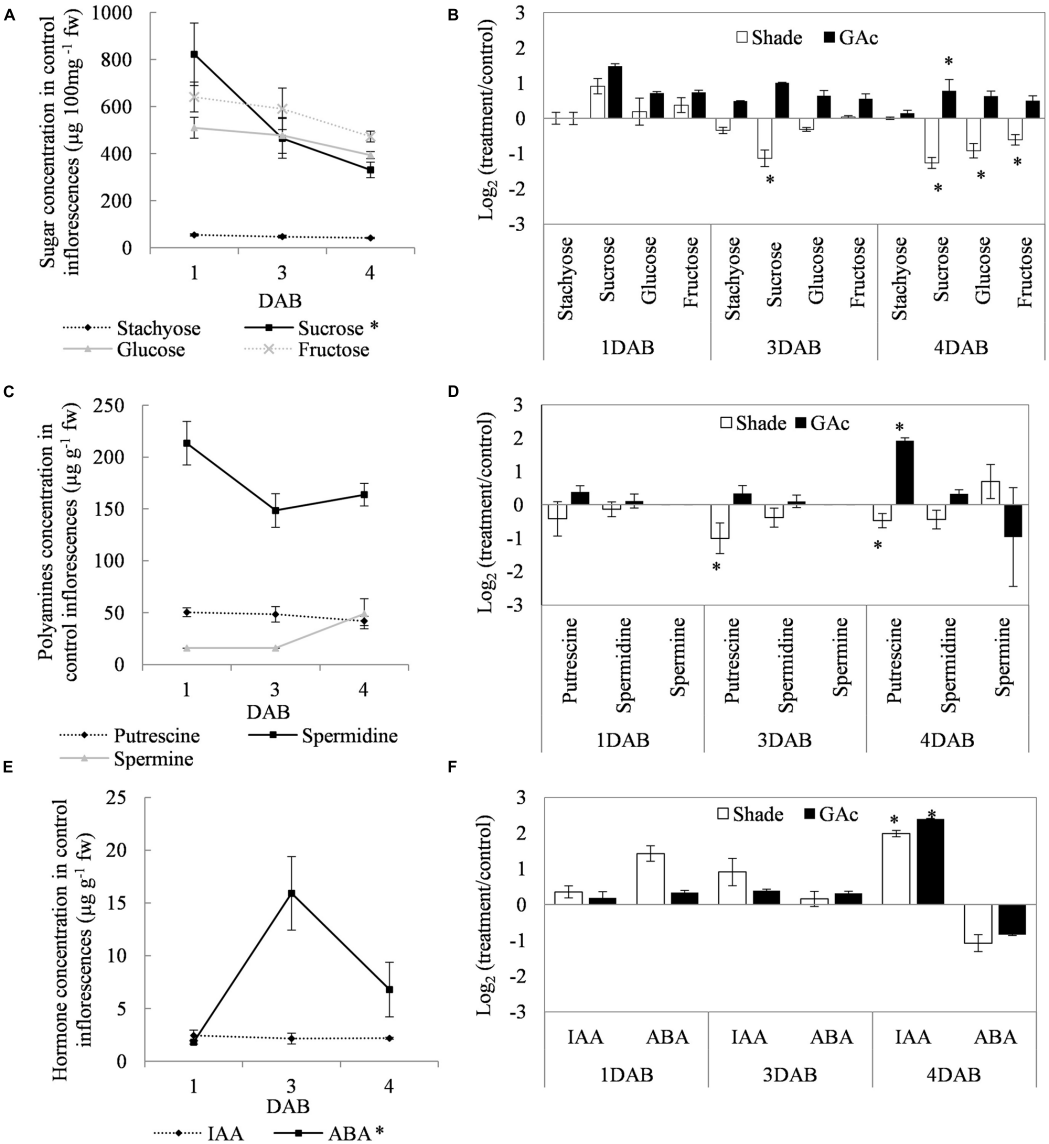
**Fluctuation in sugar **(A)**, PA **(C)**, and hormone **(E)** concentrations in control and fold-change variations [Log_2_(treatment/control)] in sugar **(B)**, PA **(D)**, and hormone **(F)** concentrations in shade and GAc treated inflorescences at 1, 3, and 4 DAB.** Statistical significances of different time points in metabolites concentration in control inflorescences, and of treatments comparing to control were assessed by one-way ANOVA (^∗^ mean significantly different at *P* < 0.05).

**FIGURE 4:**
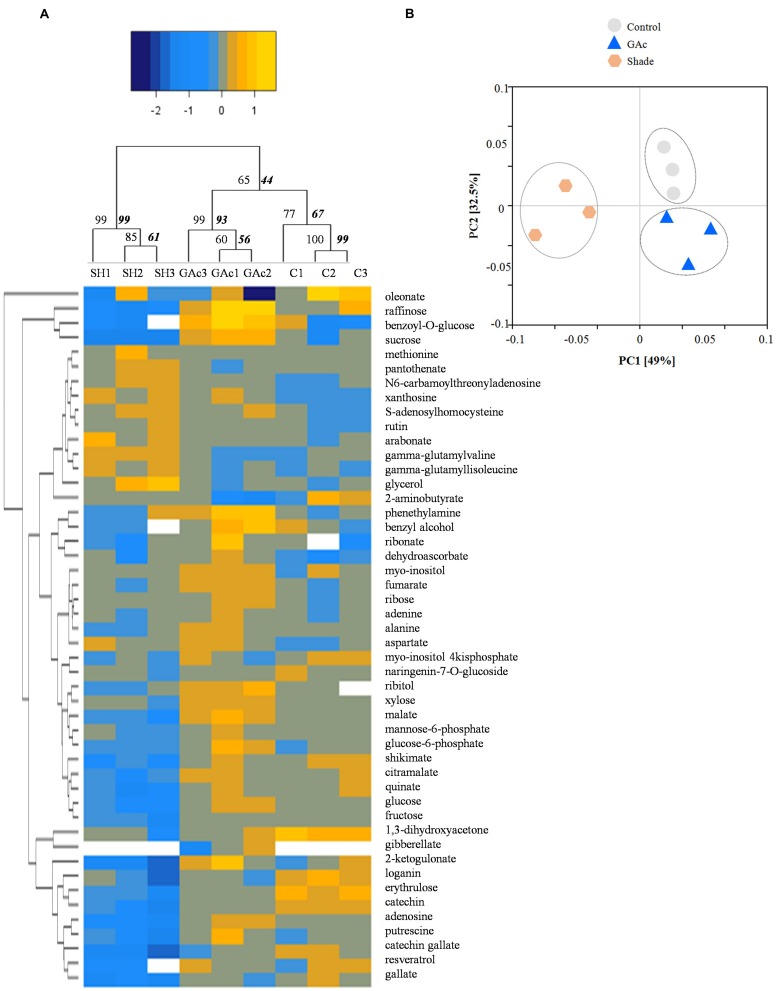
**Hierarchical cluster **(A)** and principal component analysis **(B)** of the significantly changed metabolites.** Yellow and blue tones represent metabolites more and less abundant, respectively. The significance of dendrogram nodes was estimated by bootstrap analyses using 1000 permutations. Values represented in the left side of internal nodes are the approximately unbiased *P-*values (AU), bold and italic values on the right side represented the bootstrap probability value. In PCA, the first and second components explain 81.5% of the total variation endorsed by the metabolite profile. Gray, blue and orange represent replicates from control, GAc and shade treatments, respectively.

**FIGURE 5:**
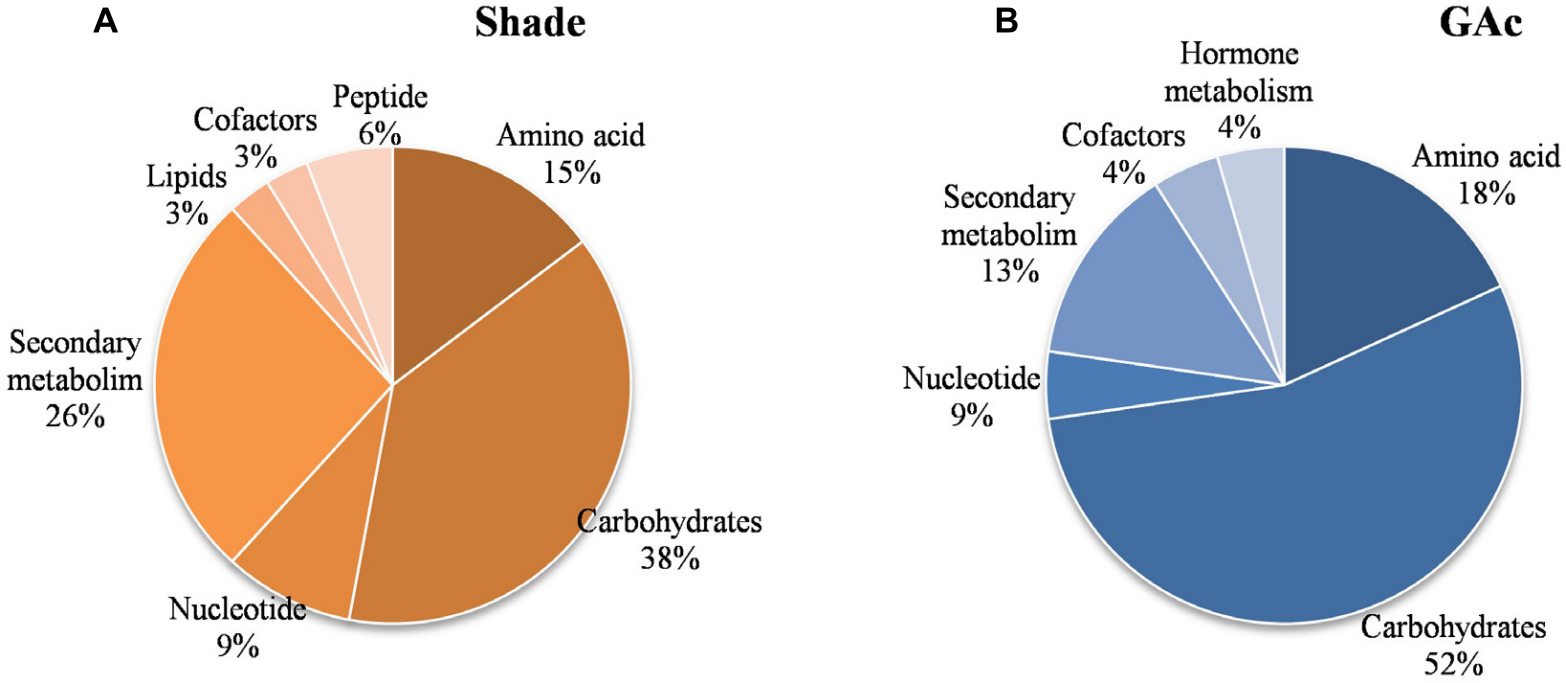
**Functional categorization of the 48 metabolites that showed significantly changes (*P* < 0.05) in abundance.** In shaded inflorescences **(A)**, 34 metabolites from carbohydrate, secondary metabolism, amino acid, nucleotide, peptide, cofactor, and lipid functional pathways were significantly affected. In GAc-treated inflorescences **(B)**, 19 metabolite contents from carbohydrate, amino acid, secondary metabolism, hormone, cofactor, and nucleotide pathways changed. Nine metabolites were changed in both the treatments.

**FIGURE 6:**
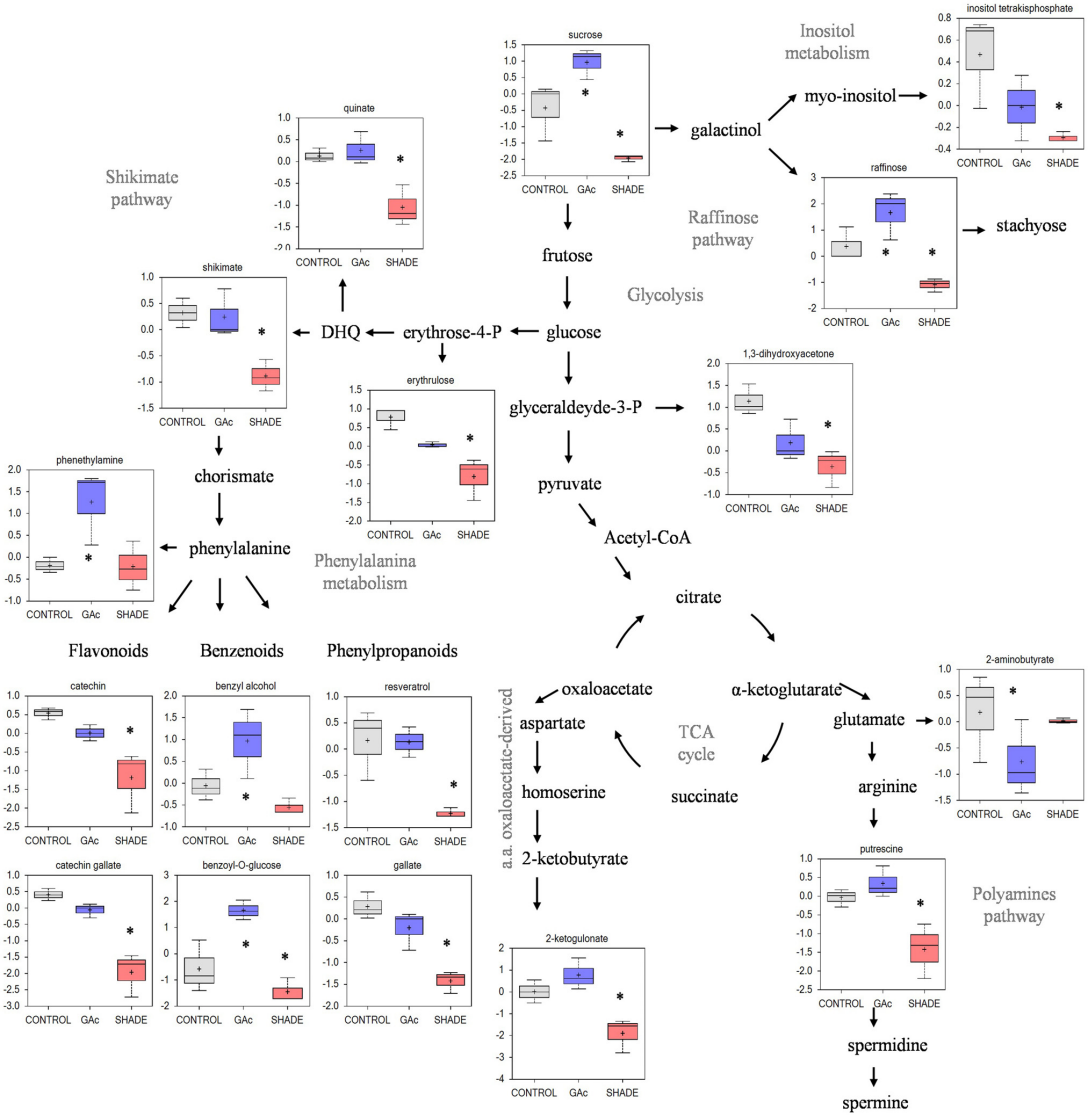
**Changes in metabolic profile in table grape inflorescences treated with GAc and Shade.** Metabolites with highly significant differences are represented in the box plots and *asterisks* identify which treatment is different from the control [*P* < 0.05, FC (| log 2 (treatment/control)| ) ≥ 1]. Data were log _2_ transformed after scale imputation median = 1. Gray, blue, and orange represent samples from control, GAc and shade treatments, respectively.

Regarding the metabolites analyzed in inflorescences sampled from untreated vines 1, 3, and 4 DAB during the late cycle, the results showed reduced sucrose levels between 1 and 4 DAB (**Figure 3A**) and increased ABA concentrations, peaking at 3 DAB (**Figure 3E**). Conversely, compared to the control, in shade-treated inflorescences, sucrose concentration decreased at 3 and 4 DAB and fructose and glucose at 4 DAB. In GAc-treated inflorescences, sucrose concentration was highest at 4 DAB (**Figure 3B**). A significant increase of putrescine content was also observed in the same samples, 4 DAB. In samples submitted to the shade treatment, this PA decreased 3 and 4 DAB (**Figure 3D**). Cadaverine was not detected. Concerning hormones, IAA concentration was significantly increased in result of both treatments 4 DAB and no differences in ABA levels were observed between treated inflorescences and controls (**Figure 3F**). From the 215 metabolites investigated by the global metabolic analyses conducted in samples collected 4 DAB, a total of 211 were detected (Supplementary Figure S2) and 48 showed to be differentially changed in abundance (*P* < 0.05) in inflorescences induced for abscission. A total of 34 and 23 metabolites showed differential abundance in shade and GAc treatments, respectively, of which 9 metabolites were common in the different treatments (**Table 3**). Hierarchical clustering (**Figure 4A**) showed the association between samples according to the metabolite profile. Samples resulting from each treatment were significantly clustered together. Oleonate, the only metabolite that highly decreased with GAc treatments (FC = -2) was separated from the other metabolites. Raffinose, sucrose and benzoyl-*O*-glucose, showed a distinct pattern according to the imposed treatment, and were grouped in a different cluster. PCA (**Figure 4B**) showed that all samples could be separated according to the treatment to each they were submitted to. The first component allows distinguishing inflorescences developing under shade from all the other samples. GAc samples were separated from controls by the second component. Differentially quantified metabolites were mapped onto general biochemical pathways, and categorized into functional classes as showed in **Figure 5**. Among the 34 metabolites significantly altered in abundance in shaded inflorescences, those assigned to carbohydrates composed the most prevalent class (38%), followed by products of secondary metabolism (26%), amino acid (15%), nucleotide (9%), peptide (7%), cofactors (3%), and lipids (3%). Among the 23 metabolites that significantly changed in response to GAc, products from carbohydrate metabolism was also the most prevalent class (52%), followed by amino acid (18%), secondary metabolism (13%), nucleotide (9%), cofactor (4%), and hormone (4%). A list of all metabolites significantly affected by GAc and shade treatments (*P* < 0.05), assigned functional categories, KEGG compound number and respective fold-change is provided in **Table 3**. Shade and GAc treatments were responsible for a decreased concentration of 24 and four metabolites, respectively, sharing two metabolites derived from the carbohydrate pathway, namely myo-inositol tetrakisphosphate and erythrulose. On the opposite trend, the imposed treatments induced increased concentration of 10 and 19 metabolites, in shade and GAc, respectively. N6-carbamoylthreonyladenosine, a metabolite from the nucleotide class, was common to both sample sets. Six metabolites concurrently increased in response to GAc and decreased under shade. Four were derived from carbohydrates metabolism, namely sucrose, glucose, raffinose, and malate and the other two were derived from secondary metabolism, and included benzyl alcohol and benzyl-*O*-glucose. Regarding amino acid pathway, decreased quinate, shikimate, and putrescine concentrations and increased metabolites derived from aspartate family (methionine and SAH) were observed in shade-derived samples. In the GAc treated samples, an increase of phenethylamine, aspartate, and alanine and a decrease of 2-aminobutyrate occurred. All metabolites from the carbohydrate pathway were reduced in shaded inflorescences except arabonate. Conversely, in GAc-treated samples, all metabolites increased except myo-inositol tetrakisphosphate and erythrulose. Glycerol, a product from the lipids metabolism, highly increased in result of shade conditions. Gibberelate was detected only in the GAc-derived samples, probably as the result of the exogenous application. Several metabolites from coenzyme and nucleotide metabolisms increased in both treatments except adenosine that was reduced in shade. Likewise, gamma-glutamylisoleucine (gamma-glu-lleu) and gamma-glutamylvaline (gamma-glu-val), from peptides metabolism, increased in the shade treatment. The concentration of metabolites derived from secondary metabolism was reduced in both treatments except two aromatic benzenoids (benzyl alcohol and benzyl-*O*-glucose) that increased in the GAc treatment and rutin that was increased in the shaded samples. Focusing on the metabolites with more pronounced changes [FC (| log 2 (treatment/control)| ) ≥ 1], it was observed that raffinose, inositol, glycolysis, TCA cycle, shikimate, PAL and PA pathways were involved in the changes that occurred in inflorescences treated to enhance abscission rates (**Figure 6**). Sucrose and raffinose amounts changed in opposite directions in shade and GAc treated inflorescences, and a down- and up-regulation of the raffinose family oligosaccharides (RFOs) pathway was found in shade and GAc, respectively. Inositol and metabolites from the shikimate pathway (quinate and shikimate) were reduced in shade. Erythrulose and 1,3-dihydroxyacetone derived from glucose and glyceraldeyde-3-P in the glycolysis pathway were also reduced in this treatment. Concerning compounds associated with the TCA cycle, 2-ketogulonate, derived from oxaloacetate, was reduced in shaded samples and 2-aminobutyrate, derived from α-ketoglutarate (via glutamate), was reduced in response to GAc spraying. PA metabolism, likewise derived from glutamate, was reduced in result of the shade treatment. Compounds from benzenoids family increased in GAc treated inflorescences whereas oleanolate was decreased. Flavonoids (catechin and catechin gallate), phenylpropanoids (resveratrol and gallate), benzyl-*O*-glucose and loganin were reduced in response to shade.

**Table 3:** List of metabolites significantly affected by GAc and shade treatments (*P* < 0.05), functional categories, KEGG compound number and respective fold-change.

Super pathway	Compound	KEGG	Log_2_ (GA_c_/control)	Log_2_ (shade/control)
Amino acid	**2-Aminobutyrate**	**C02261**	**-1.0**	
	**Phenethylamine**	**C02455**	**1.6**	
	**Quinate**	**C00296**		**-1.1**
	**Shikimate**	**C00493**		**-1.2**
	**Putrescine**	**C00134**		**-1.3**
	Alanine	C00041	0.5	
	Aspartate	C00049	0.6	
	Methionine	C00073		0.7
	*S*-adenosylhomocysteine (SAH)	C00021		0.6
Carbohydrate	**2-Ketogulonate**	**C02261**		**-1.8**
	**Ribonate**		**1.3**	
	**Raffinose**	**C00492**	**1.4**	**-1.6**
	Glucose	C00031	0.3	**-**0.9
	Glucose-6-phosphate (G6P)	C00668		**-**0.5
	Fumarate	C00122	0.8	
	Malate	C00149	0.7	**-**0.8
	Arabonate	C00878		0.8
	Ribitol	C00474	0.8	
	Xylose	C00181	0.5	
	Ribose	C00121	0.5	
	**Sucrose**	**C00089**	**1.3**	**-1.7**
	**Erythrulose**	**C02045**	**-**0.8	**-1.6**
	Fructose	C00095		**-**0.8
	Mannose-6-phosphate	C00275		**-**0.5
	Citramalate	C00815		**-**0.8
	**1,3-Dihydroxyacetone**	**C00184**		**-1.5**
	Myo-inositol	C00137	0.5	
	**Myo-inositol 4 kisphosphate (1,3,4,6/3,4,5,6/ 1,3,4,5)**	**C01272**	**-**0.7	**-1.1**
Lipids	**Glycerol**	**C00116**		**1.3**
Coenzyme	**Dehydroascorbate**	**C05422**	**1.0**	
	Pantothenate	C00864		0.7
Nucleotide	**Adenosine**	**C00212**		**-1.3**
	Adenine	C00147	0.5	
	N6-carbamoylthreonyladenosine		0.4	0.9
	Xanthosine			0.8
Hormone	**Gibberellate**	**C01699**	**3.3**	
Peptide	Gamma-glutamylisoleucine			0.7
	Gamma-glutamylvaline			0.7
Secondary metabolism	**Oleanolate**		**-2.0**	
	**Benzyl alcohol**	**C00556**	**1.1**	**-**0.8
	**Benzoyl-*O*-glucose**		**2.0**	**-1.4**
	**Catechin**	**C06562**		**-1.6**
	Naringenin-7-*O*-glucoside			**-**0.5
	Rutin	C05625		0.4
	**Catechin gallate**			**-2.3**
	**Gallate**	**C01424**		**-1.7**
	**Resveratrol**	**C01424**		**-1.9**
	**Loganin**	**C01433**		**-1.5**

Bold letters correspond to the highly significant different metabolites FC(| log 2 (treatment/control)| ) ≥ 1.

### Impacts on Bunch and Berry Quality

Shade and GAc treatments reduced yield per plant, bunch weight and number of regular sized berries, in both cycles, when compared to untreated vines (**Table 2**). In the late production cycle, no differences in shot berries number were observed while in the early cycle, GAc promoted a higher number of these berries (407.1 shot berries), which was reflected in the increased number of total berries (512.6 berries per bunch) measured. Rachis length was shorter in bunches from vines submitted to shade and GAc, in the early production cycle. Nevertheless, bunch compactness was lower in plants that were shaded during flowering in both production cycles (5.1 and 10.5 berries cm^-1^) and higher in GAc treated plants in the early production cycle (22.1 berries cm^-1^) when compared with control. Rachis weight was lower in both production cycles in bunches from shaded vines, and was higher in GAc treated bunches in late cycle. Regarding berry quality parameters, the weight and transversal diameter of the berries were reduced in grapes from GAc treated and shaded vines in late cycle, when compared with controls. In the early cycle, no significant differences were observed in berry diameter but shade lead to increased berry weight. Berry TSS content was higher in shaded vines comparing to the control in the late cycle, while differences in titratable acidity were not observed (**Table 2**). Both production cycle and the interaction production cycles × treatment significantly affected yield per plant, number of berries, number of shot berries, bunch compactness and berry diameter and berry weight (*P* < 0.05). Production cycle also affected average bunch weight and titrable acidity (*P* < 0.001).

## Discussion

### Flower Abscission Induced by Hormonal and C-Starvation Stimuli

The direct comparison of the changes in *V. vinifera* L. inflorescences metabolite abundance that resulted from the imposition of two different abscission-triggering treatments was possible due to controlled conditions allowed from the experimental model used. Using potted plants growing under soilless greenhouse conditions, it was possible to apply both treatments to homogenous biological material. Moreover, this system allowed achieving improved plant growth and grape productivity, extending the harvest schedule and, relevant to the objectives of this work, obtaining more than one production cycle in the same agronomic year (Di Lorenzo et al., 2009).

The significant effect of climatic conditions on fruit set, revealed by the differences observed in natural flower drop rates between the two production cycles, can be explained by the influence exerted by the maximum temperatures registered during bloom in the late cycle that exceeded 35°C in the majority of the days during the bloom period (Supplementary Figure S1). Under these range of temperatures, fruit set is known to decrease due to reduction of ovule fertility (Kliewer, 1977) and pollen germination rates (Vasconcelos et al., 2009).

The Black Magic table grape cultivar showed to be sensitive to shade imposed during bloom, resulting in increased flower drop percentages in both production cycles while the response to GAc application showed to be dependent of microclimate conditions. Under this treatment, fruit set was impaired in the late production cycle while an increase was observed in the early cycle, which agrees with previous results (Reynolds and Savigny, 2004; Reynolds et al., 2006) in 3-years trials. The significant reduction of fruit set induced by the 12-days period shading during bloom (**Table 1**) suggests that this approach can be exploited as an effective method for thinning in table grape production, relying on the pronounced decline of net photosynthetic rate, which promotes a decrease on carbon resources available to both vegetative and reproductive sinks and increases the competition between them (Corelli Grappadelli et al., 1990; Byers et al., 1991; Zibordi et al., 2009). The moment of shade imposition matched a stage during which the vine carbon reserves reached a minimum, which coincides with the onset of bloom in grapevines (Zapata et al., 2004). During this sensible period, interruptions or partial sugar supply declines are known to promote flower abortion (Lebon et al., 2008). In the present study, monitoring daily rate of berry drop during the shade imposition period enabled us to verify that the maximum rate of berries drop depends on the global environmental conditions, occurring 2–4 DAB in late cycle and between 4 and 12 DAB in early cycle, indicating precocity in C-shortage in the former cycle. Shading did not affect leaf area nor shoot growth, confirming previous observations that indicate that reproductive growth is more sensitive to environmental stress or limitation of resources than vegetative growth (Chiarello and Gulmon, 1991). The increased estimated leaf chlorophyll content in result from intercepted light reduction when compared with GAc treated vines, agrees with Ferree et al. (2001), and suggests an adaptability of the grapevine to low light intensity by increasing the PAR trapping efficiency (Cartechini and Palliotti, 1995).

The evidence that disturbances in growth regulators internal concentrations have an important influence on fruit set has been exploited in table grape production. In fact, GAc exogenous bloom application is commonly used as a mean to achieve cluster loosening (Dokoozlian and Peacock, 2001). Nonetheless, environment was demonstrated to play a major role in modulating the responses to growth regulator treatments, in particular the temperature. Low temperatures lead to sub-optimal response while, under high temperature conditions, the response may be excessive (Wertheim and Webster, 2005). Thus, we suggest that the observed differences on GAc effectiveness to induce flower abscission and increase shot berries number was related to the environmental conditions and physiological stage of the vines. During the late production cycle, vines are developing under more intense stress conditions, and had a smaller leaf area and shoot length than in the early cycle. Plants are expected to have lower carbohydrates and endogenous GA levels, resulting in a higher sensitivity to exogenously applied GAc and a reduction of fruit set comparing to control. Sensitivity to exogenously applied GAc was reported to be inversely related with endogenous gibberellins levels (Boll et al., 2009).

### Sugar Metabolism and Other Energy Sources

Sucrose, glucose, and fructose are the major phloem sap sugars which feed the developing vine inflorescences (Lebon et al., 2008). The reduction on the sucrose content in inflorescences developing under control conditions observed 4 DAB, at the onset of natural drop, agrees with previous observations (Glad et al., 1992) reporting that this sugar, predominant in this stage, represents 85% in sap flow at full bloom and declines thereafter to 60% at the end of fertilization, explaining natural drop. Our results were expected in confirming that decreased light intensity inhibits photosynthesis and sugar accumulation in inflorescences but showed that, in contrast, GAc treatment did not affect photosynthesis and even increased the inflorescence sugar content. Noticeably, both treatments resulted in similar rates of flower abscission (**Table 1** and **Figure 3**). Shade induced more pronounced effects than GAc spraying concerning the number of changed metabolites (**Table 3**). Essentially, all carbon metabolites identified showed to be present in lower amounts in shaded and in higher levels in GAc samples, including sucrose and glucose, as well as TCA intermediates (malate, citramalate, and fumarate), and intermediates of the RFO pathway, such as raffinose (**Table 3** and **Figure 6**). The decline of carbohydrate transport metabolism that occurred in shade agrees with abscission modulation induced by NAA and by shade in apple (Zhu et al., 2011). It was also verified that under shade, as in other stress conditions, the synthesis of glycerol may be favored via starch degradation, as an energy resource, and decrease of the carbon flow into TCA cycle (Xia et al., 2014). Regarding amino acid pathways, in shaded samples, the concentration of quinate, shikimate, and putrescine decreased while methionine and SAH increased (**Table 3** and **Figure 6**). In addition, adenosine, which plays an important role in biochemical processes as energy transfer [adenosine triphosphate and diphosphate (ATP and ADP) and in signal transduction cyclic adenosine monophosphate (cAMP)], also decreased. Shade conditions led to a signature of carbon/nitrogen (C/N) imbalance with lower energy and carbon metabolites, biosynthetic precursors such as shikimate and nitrogen-rich compounds associated with anabolic activity such as putrescine, and higher proteinogenic amino acid such as methionine that may result from protein turnover to free up amino acid carbon backbones for energy utilization. Likewise, the increased amount of pantheonate (vitamin B_5_) whose biosynthetic pathway involves valine and alanine amino acids (Raman and Rathinasabapathi, 2004) observed in shade-derived inflorescences support the hypothesis of proteinogenic amino acids abundance from protein turnover. On the other hand, since all Calvin cycle metabolites were present in lower amounts in shaded samples, the pathway of pantheonate functioning as CoA biosynthesis precursor needs a more detailed evaluation. Our results are in accordance with Baena-González and Sheen (2008) which review physiological and molecular responses associated with plant energy deficit, including activation of catabolic pathways to provide alternative nutrient, metabolite and energy sources, and a decline in the activity of biosynthetic enzymes to preserve energy, and with Aziz (2003) showing that shading the vines at full bloom causes a decrease in both sugars and free PAs and leads to a substantial increase of abscission.

Gibberellins are involved in pathways of regulation of flowering and fruit-set in grapes, as active GAs, mainly GA_1_, peaks at anthesis and decrease thereafter (Perez et al., 2000; Giacomelli et al., 2013). GAc is commonly applied during bloom to reduce fruit set but the molecular mechanisms underlying this process are largely unknown. In *Arabidopsis thaliana*, GAc induces increased 3-*P*-glycerate and promotes plant growth rate (Meyer et al., 2007; Ribeiro et al., 2012). In this study, GAc application led to generalized up-regulation of both primary (carbohydrates, amino acid, coenzyme, and nucleotide pathways) and secondary metabolisms (**Table 3** and **Figure 6**). Since no changes in photosynthetic rate (source) were detectable in samples submitted to this treatment, we hypothesize that an increase on inflorescences sink strength occurs after GAc treatments, resulting in the formation of king berries, with higher potential to compete for carbohydrates and other metabolites and higher growth rate, inhibiting the development and inducing abscission of later flowers. Regarding TCA cycle-derived metabolites, only 2-aminobutyrate from glutamate family decreased as a result of GAc while, on the other hand, metabolites derived from aromatic amino acid phenylalanine and from aspartate family (alanine and aspartate) showed the opposite trend (**Table 3** and **Figure 6**). Glutamate derives from α-ketoglutarate and can be involved in the biosynthesis of 2-aminobutyrate or, alternatively, in the biosynthesis of arginine and PAs biosynthesis. According to our results, it can be hypothesized that the pathway from glutamate to PAs is favored when vines are treated with GAc, in contrast with biosynthesis of 2-aminobutyrate.

### Cell Wall Modifications

The recorded increase of CW monosaccharides in samples from both abscission-triggering treatments (**Table 3**) sounds with the known CW disassembly and remodeling processes that occur during pedicel AZ formation as part of the coordinated series of modifications that ultimately lead to CW loosening, cell separation and differentiation of a protective layer on the proximal side after organ detachment (Lee et al., 2008). The increased arabonate concentration, which is a metabolite derived from arabinose, as consequence of the shade treatment contrasts with the observations in GAc treated inflorescences where xylose was the increased monosaccharide (**Table 3**). These differences are likely to reflect differences on target CW polymers, with pectins and xyloglucans more affected by shade or GAc, respectively. Pectin changes depends on the type of substitutions and branches in their backbone and are considered a central event (Fukuda et al., 2013) since the continuity between AZ cells is preserved by the middle lamellae, which is rich in this class of polymers, responsible for cell–cell adhesion. Pectins are additionally responsible for modulating the CW porosity and, in so, controlling the enzymes access to their substrates (Baron-Epel et al., 1988). Augmented arabinose levels may also indicate a higher substitution of pectic polysaccharides with arabinosyl residues which can work as plasticizers (Harholt et al., 2010) and be involved in the formation of the protective layer in the proximal area. In fact, during abscission, CWs of the proximal area are relatively richer in cellulose, arabinose-rich polymers and pectin, and poorer in xylan-rich polysaccharides and lignin when compared with AZ CWs (Lee et al., 2008). Regarding the detection of increased concentrations of xylose in samples from GAc-treated inflorescences, it may similarly reflect CW loosening processes needed for organ shed or CW strengthening requirements, but through action on cellulose-xyloglucan contact points. Xyloglucans are closely intertwined with cellulose at limited sites designed as “biomechanical hotspots,” promoting selective targets majorly modulating CW loosening (Park and Cosgrove, 2012). Our results confirm the putative role of xyloglucans in providing CW strength for attachment of organs and its dynamic metabolism in mediating abscission, in response to some triggering signals. These assumptions are further supported by gene expression assays since it has been demonstrated that the activation of the abscission molecular machinery involves alterations of genes encoding CW remodeling enzymes acting on structural polysaccharides leading to the middle lamellae breakdown, accompanied by distortion and dissolution of primary CWs along the abscission plane (Lashbrook and Cai, 2008; Lee et al., 2008; Agusti et al., 2009; Meir et al., 2010; Zhu et al., 2011; Peng et al., 2013; Wang et al., 2013) and glycosyl hydrolysis (Lashbrook and Cai, 2008; Singh et al., 2011, 2013). The pattern of differential temporal regulation of distinct classes of CW-related genes (Lashbrook and Cai, 2008) additionally suggests that the differences observed between treatments may be the result of triggering their action at different stages of the process. It should be noted that the samples here investigated include cells other than AZ. Hence, as CWs represent primarily communication between the plant and the environment, a role in adaptation to the imposed abiotic stress can be discussed. The observed difference in CW composition are known to be related to events such as localized cell division, arrestment of elongation and modifications in the differentiation status, to impact anatomy and development (Braidwood et al., 2013).

### Markers of Oxidative Stress

Likewise, both abscission-triggering stimuli lead to oxidative stress related metabolism, but the results suggest that different pathways are tracked. Some of the significant increases observed are related to metabolites associated with oxygen stress remediation. Gamma-glutamyl amino acids, observed in shaded samples, are intermediates in the glutathione synthesis cycle (**Table 3**) and dehydroascorbate, observed in GAc treated samples, indicates responses to elevated oxidative stress conditions related to the ROS scavenging coupled ascorbate/dehydroascorbate cycle (**Table 3**). During abscission a continuous increase of ROS production is known to occur. ROS role in abscission encompasses multiple steps of signaling (Sakamoto et al., 2008) associated with ROS-sugar-hormone cross talk (Botton et al., 2011) and ROS-mediated oxidative damage/cleavage on CW components leading to cell separation (Cohen et al., 2014). Regulation of excessive ROS by the free radical scavenging systems comprises essential enzymatic components and non-enzymatic molecules such as ascorbate and glutathione. Glutathione and ascorbate play important roles individually or through the ascorbate glutathione cycle, having specific functions besides interchangeable antioxidants (Bohnert and Sheveleva, 1998). Our results suggest that distinct metabolite-dependent responses are triggered by each treatment agreeing with the independence and interdependence of glutathione and ascorbate in peroxide metabolism model proposed by Foyer and Noctor (2011).

### Hormone Regulation

The occurrence of an ABA peak 3 DAB in control inflorescences (**Figure 3E**) preceding the rise of natural flower drop (4–12 DAB; **Figure 2A**) is in accordance with previous works describing hormones as mediators of the AZ cell responses to abscission signals (Estornell et al., 2013). Interplay between a decrease of sugar, increases levels of ABA, ethylene, and ROS in organ predicted to abscise were verified, taking place before the onset of abscission (Botton et al., 2011). Our results confirm ABA as a component of the self-regulatory mechanism that adjusts fruit load to carbon supply occurring under natural conditions or following treatments (Gomez-Cadenas et al., 2000).

The increase of inflorescence IAA (auxin) concentration registered in both treatments (**Figure 3F**) may suggest that IAA was accumulated on the proximal side of abscission and the auxin flux to the distal organ predict to abscise was interrupted. It has been showed that a constant auxin transport through the AZ is needed to prevent abscission (Taylor and Whitelaw, 2001) and a auxin depletion linked with acquisition of ethylene sensitivity within AZ cells is needed to its induction (Meir et al., 2010; Basu et al., 2013). Our results are also consistent with the auxin gradient theory (Addicott et al., 1955) based on the evidence that auxin application in the proximal end of AZ explants accelerates abscission whereas when applied at the distal end delays it, and suggesting that changes in auxin gradients may act in signaling the onset of senescence and abscission. Ethylene and auxins are critical factors that regulate the onset of abscission (Basu et al., 2013) in a mechanism where the auxin depletion inside AZs and an altered expression of auxin-regulated genes induce the acquisition of sensitivity to ethylene and AZ activation. The increase of methionine and SAH, which are intermediates in the ethylene biosyntheses, observed in shaded-treated samples (**Table 3**) can be associated with the increase of ethylene, acting as a trigger in the abscission process (Meir et al., 2010). SAM, derived from methionine, is also the precursor of the spermidine and spermine biosynthesis pathway or alternatively can be used on the synthesis of ACC which is the immediate precursor of ethylene (Wang et al., 2002).

### Secondary Metabolism

In shade, decreased loganin content, which is a monoterpenoid intermediate in the production of indole alkaloids, and several phenylpropanoids, benzenoids, and flavonoids was observed (**Table 3** and **Figure 6**), indicating suppression of biosynthesis of secondary metabolites and a slowdown of biochemical reactions in the AZ and neighboring tissues (Wang et al., 2013). This significant reduction can also mean an initial delay in fruit set and development under these conditions due to drastic reductions in carbon supply during this period, when compared to control samples. In this later situation, the accumulation of compounds characteristics of berry development, mainly in red and black varieties as ‘Black Magic,’ is known to be already started (Braidot et al., 2008). The decreased catechin can be also the result of the condensation of such flavanols, as observed after ethylene exogenous application (Rizzuti et al., 2015). Among the metabolites analyzed, flavonoid rutin was the exception in the general trade, showing a slightly increase in shade, probably due to its potential as strong radical scavenger and inhibitor of lipid peroxidation (Kumar and Pandey, 2013). On the other hand, GAc application led to a general advance in berry development in this stage and can have the opposite effect in ripening, depending on the cultivar (Teszlák et al., 2005). Comparing to the control, the aromatic compounds (benzenoids) showed increased accumulation in GAc treated samples (**Table 3**). Also in GAc, the decreased terpenoid oleonate levels measured suggests a reduction of steroids synthesis, which are membrane components that appears to control membrane fluidity and permeability and, in some plants, have a specific function in signal transduction (Piironen et al., 2000).

### Final Development of Reproductive Structures

The treatments imposed to produce biological samples enriched in abscission signals affected final yield and quality in both production cycles and some implications can be ascertain with relevance for table grape production (**Table 2**). The reduction of the number of berries in the late production cycle in shade and GAc treatments lead to reductions of bunch weight and yield per plant, indicating that the two approaches were efficient in inducing abscission. However, in the late cycle, both treatments affected berry weigh and diameter in a detrimental way, which can be the result of a decreased seed number and weight (Reynolds et al., 2006). In early cycle, shade resulted in a successful thinning method reducing total berries number and improving berry weight and diameter. The shade treatment affected bunch characteristics, reducing rachis length and weight and still reducing the number of berries per centimeter of rachis, in both cycles. The observed effect on the rachis can result from competition for photoassimilates favoring vegetative growth in detriment of the development of reproductive organs (Chiarello and Gulmon, 1991). In the early cycle, GAc treatment showed to be ineffective as thinning method due to the increased number of shot berries, total berries number and bunch compactness. The high number of shot berries observed as a negative effect of GAc application was also described by Dokoozlian and Peacock (2001). Recently, in a study performed by Abu-Zahra and Salameh (2012) aimed at evaluating the impact of GAc spray (50 ppm at the end of bloom, 18 and 40 days after end of bloom) on ‘Black Magic’ grape quality, an increase of berries number, berry size, TSS, titratable acidity, and decreased color intensity was observed. Although, according to Cartechini and Palliotti (1995), shade during flowering has no effect on final berries sugar content, under our conditions, the shade treatments increased TSS in the late production cycle, which can be a direct result of the reduction of berries weight and diameter.

### A Mechanistic View of Flower Abscission Control in *Vitis vinifera* L.

**FIGURE 7:**
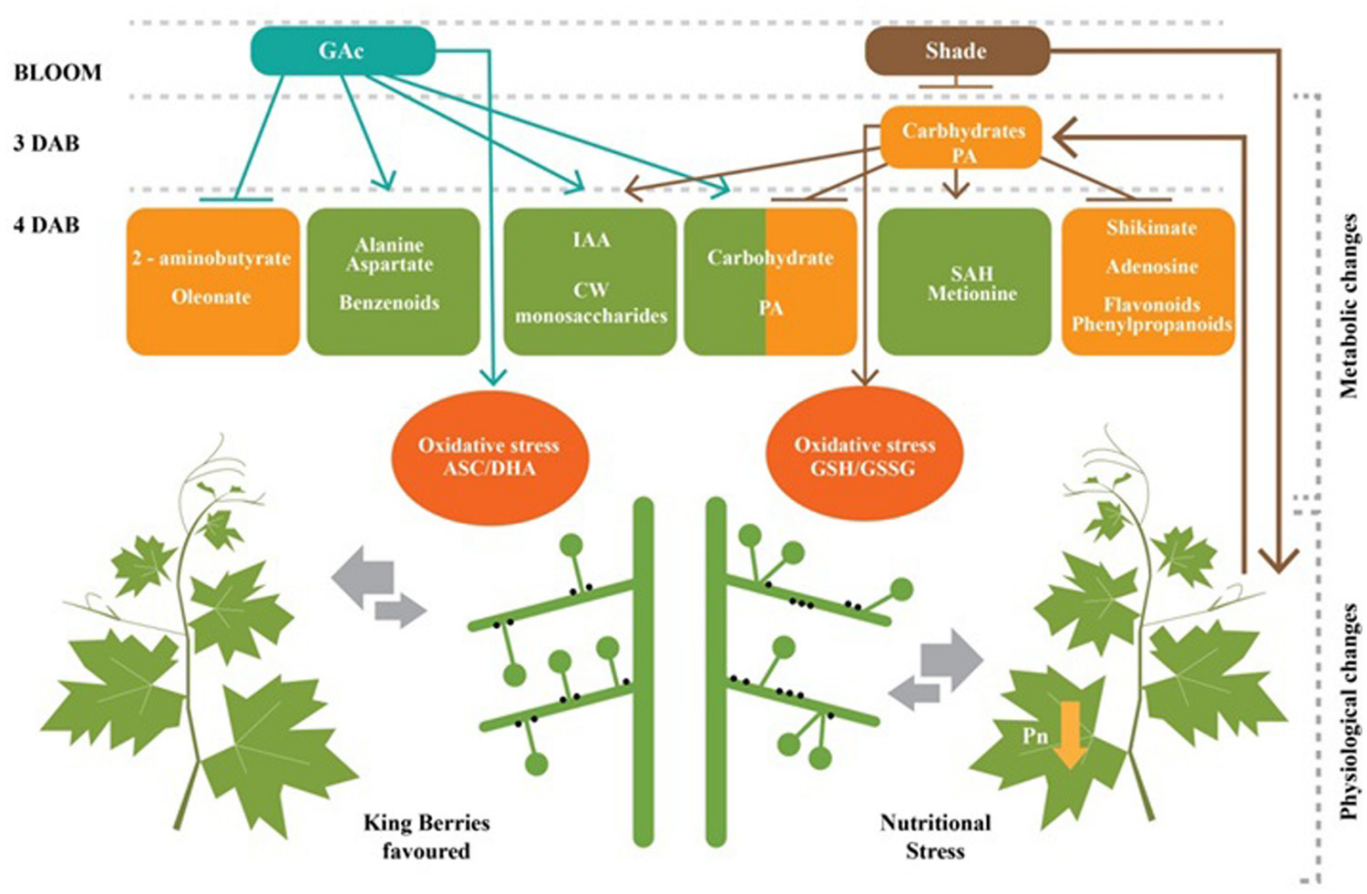
**A proposed mechanistic model for flower abscission in *Vitis Vinifera* L. inflorescences triggered by GAc and shade.** Shade treatment reduced net photosynthetic rate which lead to significant alterations 3 and 4 DAB, including global carbohydrates starvation, reduction on shikimate, PA (putrescine) and secondary metabolisms and increase of oxidative stress, revealed by glutathione remediation cycle. GAc induced an increase on carbohydrates, PA (putrescine), amino acids and secondary metabolisms and oxidative stress, revealed by ascorbate/dehydroascorbate remediation couple at 4 DAB. Both treatments induced IAA and CW monosaccharide accumulation. The thickness of the arrows related to inter-organ competition is proportional to the sink strength at bloom stage. According to this model, flower abscission in shade is due to a general nutritional stress and, in GAc treatment to the growth of king berries which inhibits the development of lateral flowers. Abscission layer in plant side is represented by black dots. Green and orange boxes indicate the increase and decrease on metabolite concentrations, respectively, as response of imposed treatments.

The analysis performed in the late cycle, when both treatments were efficient in inducing abscission, showed that GAc responses comprised a relatively low numbers of significant changes, while the shade treatment conduced to more dramatic physiological and metabolomic alterations (**Table 3**). These results allowed us to propose a mechanistic model to explain differences and common links for flower abscission determination in response to two stimuli (**Figure 7**). Comparing the composition in metabolites of grapevine inflorescences treated with the different abscission inducers (shade and GAc) and the control, we can conclude that abscission mechanisms triggered by hormonal application and via C-starvation are not based in the same pathways. A new insight on the mode of action of GAc during bloom is here provided, showing that it is based on a generally stimulation of cell metabolism and gene expression revealed by ribose and its derived metabolites, sugar, amino acid and PA metabolisms in the whole inflorescence and a highly significant inhibition of a glutamate sub-pathway related with 2-aminobutirate (**Table 3**), which can be a key step in the GAc metabolism in inflorescences at bloom stage and may participate in a cross talk between IAA and gibberellate. In other biological processes (Yamaguchi, 2008), it has been described that bioactive gibberellins and auxins can positively regulate flower abscission triggered by GAc spraying. On other hand, shade induced abscission through energy deprivation mechanisms showed by the decline of photosynthesis, carbon metabolism and biosynthetic activity. The increased accumulation of ethylene precursors suggests that these events may participate together with ethylene production (**Table 3**). The common markers of abscission were increased IAA concentration in inflorescences, which can be a result of an auxin gradient change through the AZ, and increases in oxidative stress marker metabolites agreeing with previous studies in other species (Estornell et al., 2013). Despite the grapevine economic value and scientific relevance as a model species, this study provides the first mechanistic view of the metabolomic changes responsible for the flower abscission regulation in this species (**Figure 7**), triggered by exogenous GAc application and reduction of the intercepted light, unraveling the complexity of its opposite effects and contributing to the advance in knowledge that will ultimately may lead to improved control of grapevine fruit set.

## Author Contributions

SD, PS, CO, and LG were responsible for the conception and design of the experiments, SD and PS were responsible for acquisition of data, SD, VC, and AL performed the laboratory analyses, SD, PS, RD, CO, and LG interpreted the data. All authors drafted and approved the manuscript. RD, CO, and LG steered the whole project.

## Conflict of Interest Statement

The authors declare that the research was conducted in the absence of any commercial or financial relationships that could be construed as a potential conflict of interest.

## Footnotes

1 www.genome.jp/kegg/

2 www.plantcyc.org/

## Abbreviations

ABA: abscisic acid
ACC: 1-aminocyclopropane-1-carboxylic acid
CW: cell wall
DAB: days after bloom
E: transpiration rate
GAc: gibberellic acid
*gs*: stomatal conductance
IAA: indolacetic acid
PAs: polyamines
PAL: phenylalanine
Pn: net photosynthetic rate
ROS: reactive oxygen species
SAH: *S*-adenosylhomocysteine
SAM: *S*-adenosylmethionine
TCA: tricarboxylic acid
