# Supplementary material for: Flower abscission in Vitis vinifera L. triggered by gibberellic acid and shade discloses differences in the underlying metabolic pathways
Source: Front Plant Sci. 2015 Jun 22;6:457. doi: 10.3389/fpls.2015.00457 (PMC4476107; doi:10.3389/fpls.2015.00457)
Supplement: Supplementary file 1 [file Image_1.PDF]

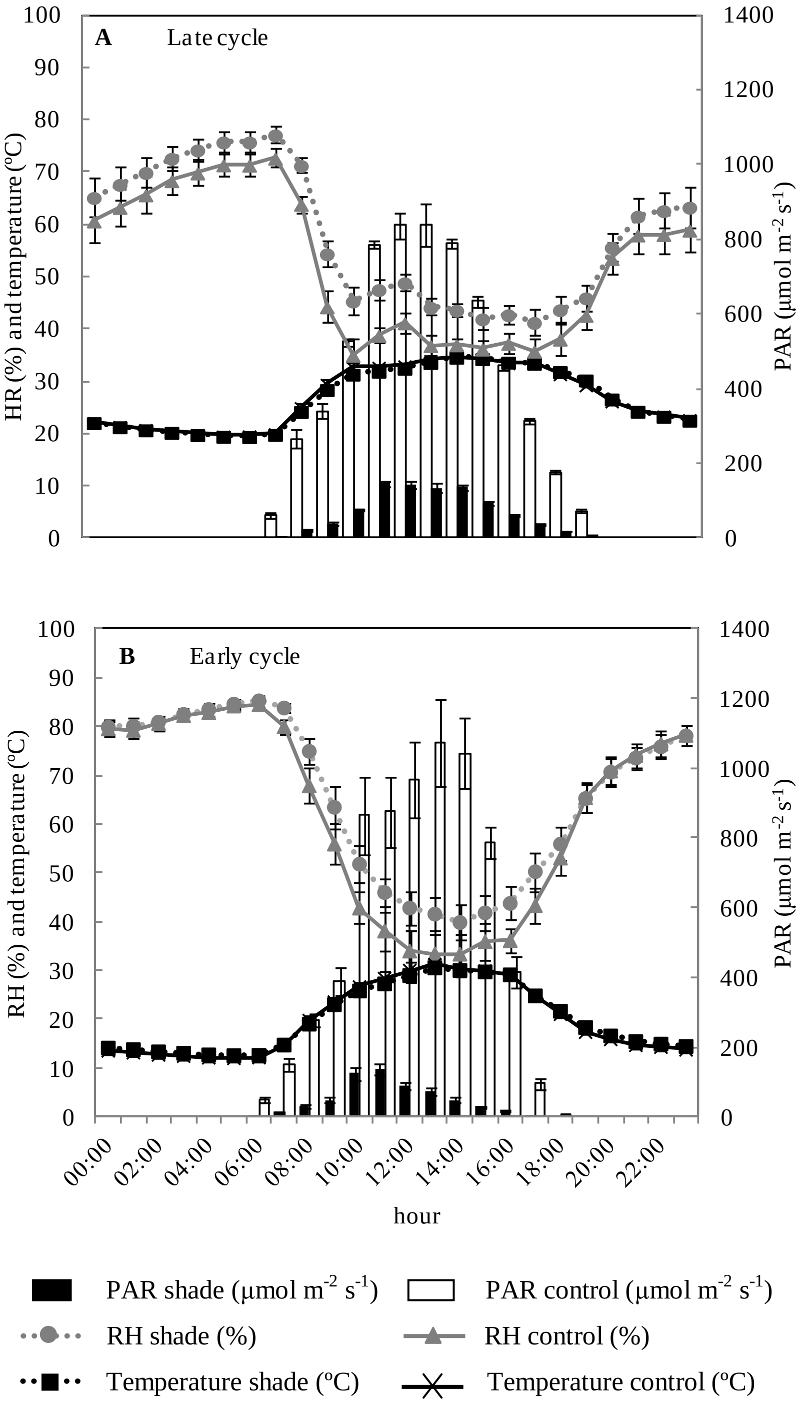

**C** DAB	Maximum temperature (ºC)
1	37.5
2	36.6
3	37.1
4	35.8
5	35.8
6	33.2
7	33.2
8	32.4
9	34.5
10	35.3
11	36.2
12	35.3

**D** DAB	Maximum temperature (ºC)
1	32.4
2	31.6
3	32.0
4	34.1
5	32.0
6	34.5
7	29.6
8	26.8
9	28.7
10	30.3
11	37.9
12	29.2

Figure S1. Microclimate conditions recorded during bloom period (twelve days) under shaded and unshaded conditions in late (A) and early (B) production cycles. Mean values per hour of relative humidity (RH), temperature and photosynthetic active radiation (PAR) (mean±se). Maximum temperatures registered in late (C) and early (D) cycle during the same period.
