# Supplementary material for: Flower abscission in Vitis vinifera L. triggered by gibberellic acid and shade discloses differences in the underlying metabolic pathways
Source: Front Plant Sci. 2015 Jun 22;6:457. doi: 10.3389/fpls.2015.00457 (PMC4476107; doi:10.3389/fpls.2015.00457)
Supplement: Supplementary file 2 [file Image_2.PDF]

Figure S2. Heat map illustrating metabolite changes in grapevine inflorescences in response to GAc and shade treatments. Red and green shaded cells indicate P < 0.05 (red indicates that the mean values are significantly lower for that comparison; green values significantly higher). Light red and light green shaded cells indicate 0.05 ≤ P ≤ 0.10 (light red indicates that the mean values trend lower for that comparison; light green values trend higher).

				GAc/ Control	Shade/ Control	GAc/Control		Shade/Control	
**Super Pathway**	**Sub Pathway**	**Biochemical Name**	**KEGG**	**$Log_2(FC)$**	**$Log_2(FC)$**	**p-value**	**q-value**	**p-value**	**q-value**
Amino acid	Serine family (phosphoglycerate derived)	beta-hydroxypyruvate	C00168	**0.65**	0.42	0.050	0.434	0.154	0.425
		betaine	C00719	-0.07	0.28	0.912	1.000	0.139	0.425
		glycine	C00037	0.20	0.18	0.619	0.888	0.741	0.847
		serine	C00065	**0.52**	-0.09	0.099	0.493	0.759	0.853
		S-methylmethionine	C05319	0.16	0.29	0.718	0.949	0.362	0.635
		S-methylcysteine		0.00	0.44	1.000	1.000	0.603	0.776
	Aromatic amino acid metabolism (PEP derived)	dihydoxyphenylalanine (L-DOPA)	C00355	-0.10	-0.06	0.661	0.910	0.879	0.894
		phenylalanine	C00079	**-0.49**	**0.45**	0.084	0.493	0.059	0.307
		quinate	C00296	0.15	**-1.12**	0.746	0.951	0.006	0.064
		shikimate	C00493	-0.04	**-1.22**	0.751	0.951	0.000	0.005
		tryptophan	C00078	**-0.32**	0.20	0.091	0.493	0.248	0.530
		tyrosine	C00082	-0.30	-0.25	0.476	0.785	0.640	0.793
		tyramine	C00483	0.58	0.11	0.261	0.662	0.859	0.894
		phenethylamine (isobar with 1-phenylethanamine)	C02455	**1.59**	0.03	0.002	0.084	0.939	0.915
	Aspartate family (OAA derived)	2-aminoadipate	C00956	-0.67	-0.01	0.235	0.662	0.694	0.831
		2-oxoadipate	C00322	**-1.18**	0.15	0.096	0.493	0.476	0.720
		alanine	C00041	**0.45**	-0.15	0.024	0.325	0.366	0.635
		asparagine	C00152	-0.60	0.88	0.263	0.662	0.155	0.425
		aspartate	C00049	**0.59**	0.41	0.049	0.434	0.158	0.426
		glutarate (pentanedioate)	C00489	**-0.97**	0.01	0.099	0.493	0.980	0.915
		homoserine	C00263	0.26	-0.32	0.438	0.770	0.643	0.793
		lysine	C00047	0.55	0.20	0.154	0.569	0.543	0.755
		methionine	C00073	0.10	**0.66**	0.616	0.888	0.007	0.073
		methionine sulfoxide	C02989	-0.47	0.08	0.132	0.568	0.709	0.831
		pipecolate	C00408	0.39	0.29	0.375	0.714	0.421	0.708
		S-adenosylhomocysteine (SAH)	C00021	0.46	**0.60**	0.104	0.502	0.036	0.220
		threonine	C00188	-0.01	-0.29	0.956	1.000	0.146	0.425
		6-oxopiperidine-2-carboxylic acid		-0.81	-0.06	0.151	0.569	0.882	0.894
		2-hydroxyadipate	C02360	-0.71	0.65	0.227	0.662	0.145	0.425
	Glutamate family (alpha-ketoglutarate derived)	1,3-diaminopropane	C00986	-0.47	0.15	0.157	0.569	0.863	0.894
		2-aminobutyrate	C02261	**-0.97**	-0.30	0.033	0.390	0.696	0.831
		4-acetamidobutanoate	C02946	-0.04	-0.27	0.892	1.000	0.332	0.622
		4-hydroxybutyrate (GHB)	C00989	0.42	0.45	0.543	0.844	0.589	0.776
		arginine	C00062	-0.56	-0.54	0.478	0.785	0.753	0.851
		carboxyethyl-GABA		-0.30	0.36	0.140	0.569	0.142	0.425
		gamma-aminobutyrate (GABA)	C00334	0.58	0.23	0.256	0.662	0.605	0.776
		glutamate	C00025	-0.14	-0.18	0.415	0.749	0.325	0.622
		glutamine	C00064	0.11	0.34	0.720	0.949	0.251	0.530
		histidine	C00135	0.31	0.85	0.656	0.910	0.170	0.440
		N-acetylproline		-0.36	0.58	0.347	0.690	0.179	0.456
		N-acetylputrescine	C02714	0.03	-0.34	0.861	1.000	0.193	0.463
		proline	C00148	0.69	0.23	0.117	0.541	0.612	0.776
		pyroglutamine		0.10	0.04	0.826	1.000	0.885	0.894
		stachydrine	C10172	-0.36	0.30	0.173	0.569	0.164	0.434
		trans-4-hydroxyproline	C01157	**0.60**	0.10	0.089	0.493	0.833	0.894
		homostachydrine	C08283	-0.04	-0.04	0.838	1.000	0.838	0.894
		N-delta-acetylornithine		0.19	-0.22	0.529	0.839	0.342	0.622
		4-guanidinobutanoate	C01035	0.25	0.01	0.174	0.569	0.951	0.915
	Branched Chain Amino Acids (OAA derived	isoleucine	C00407	-0.29	-0.10	0.385	0.720	0.880	0.894

	Branched Chain Amino Acids (pyruvate derived)	2,3-dihydroxyisovalerate	C04039	0.19	-0.15	0.815	1.000	0.433	0.709
		leucine	C00123	-0.36	-0.30	0.279	0.662	0.433	0.709
		levulinate (4-oxovalerate)		-0.49	-0.23	0.328	0.690	0.609	0.776
		valine	C00183	-0.25	0.03	0.332	0.690	0.885	0.894
		2-isopropylmalate	C02504	-0.20	-0.40	0.444	0.770	0.206	0.481
	Amines and polyamines	5-methylthioadenosine (MTA)	C00170	0.34	**0.75**	0.531	0.839	0.075	0.342
		putrescine	C00134	0.40	**-1.29**	0.238	0.662	0.000	0.006
		spermidine	C00315	-0.42	**-0.94**	0.476	0.785	0.070	0.327
	Glutathione metabolism	5-oxoproline	C01879	-0.03	0.69	0.886	1.000	0.121	0.403
		glutathione, oxidized (GSSG)	C00127	0.11	0.10	0.637	0.902	0.689	0.831
Carbohydrate	Glycolysis	1,3-dihydroxyacetone	C00184	**-0.92**	**-1.47**	0.064	0.462	0.006	0.064
		fructose-6-phosphate	C05345	0.28	-0.38	0.448	0.770	0.239	0.523
		glucose	C00031	**0.34**	**-0.86**	0.035	0.390	0.000	0.002
		glucose-6-phosphate (G6P)	C00668	**0.39**	**-0.54**	0.091	0.493	0.018	0.125
		glycerate	C00258	0.41	-0.58	0.283	0.662	0.102	0.378
		Isobar: fructose 1,6-diphosphate, glucose 1,6-diphosphate, myo-inositol 1,4 or 1,3-diphosphate		-0.30	-0.47	0.278	0.662	0.101	0.378
		pyruvate	C00022	0.29	-0.27	0.177	0.569	0.182	0.456
	TCA cycle	alpha-ketoglutarate	C00026	-0.04	**-0.79**	0.617	0.888	0.083	0.364
		citrate	C00158	**0.30**	-0.20	0.081	0.493	0.266	0.533
		fumarate	C00122	**0.80**	0.16	0.001	0.043	0.458	0.710
		isocitrate	C00311	**-0.25**	-0.10	0.054	0.434	0.454	0.710
		malate	C00149	**0.74**	**-0.79**	0.000	0.005	0.000	0.002
		succinate	C00042	0.12	0.03	0.595	0.886	0.966	0.915
	Calvin cycle and pentose phosphate	sedoheptulose-7-phosphate	C05382	0.59	0.16	0.407	0.741	0.606	0.776
	Photorespiration	glycolate (hydroxyacetate)	C00160	0.26	-0.20	0.389	0.720	0.436	0.709
		tartarate	C00898	-0.06	**0.30**	0.744	0.951	0.097	0.378
	Amino sugar and nucleotide sugar	2-ketogulonate	C15673	0.82	**-1.84**	0.228	0.662	0.005	0.064
		arabonate	C00878	-0.01	**0.77**	0.986	1.000	0.015	0.109
		erythritol	C00503	0.38	0.25	0.105	0.502	0.220	0.502
		erythronate		-0.06	-0.15	0.753	0.951	0.283	0.563
		gluconate	C00257	0.49	-0.32	0.175	0.569	0.328	0.622
		ribitol	C00474	**0.82**	-0.10	0.004	0.100	0.875	0.894
		ribonate		**1.34**	0.10	0.013	0.240	0.738	0.847
		ribose	C00121	**0.52**	0.03	0.003	0.084	0.821	0.894
		ribulose	C00309	0.37	0.19	0.337	0.690	0.753	0.851
		threitol	C16884	0.36	0.06	0.341	0.690	0.814	0.894
		xylonate	C05411	0.19	0.10	0.163	0.569	0.542	0.755
		xylose	C00181	**0.55**	-0.27	0.003	0.084	0.105	0.378
		xylulose	C00310	0.41	-0.76	0.915	1.000	0.168	0.439
	Inositol metabolism	chiro-inositol		0.36	0.26	0.432	0.767	0.463	0.710
		inositol 1-phosphate (I1P)	C04006	0.34	-0.10	0.153	0.569	0.643	0.793
		myo-inositol	C00137	**0.50**	-0.06	0.022	0.309	0.937	0.915
		myo-inositol hexakisphosphate	C01204	**-1.15**	-0.71	0.055	0.434	0.105	0.378
		scyllo-inositol	C06153	0.45	0.26	0.254	0.662	0.368	0.635
		myo-inositol tetrakisphosphate (1,3,4,6 or 3,4,5,6 or 1,3,4,5)	C01272	**-0.74**	**-1.09**	0.016	0.257	0.002	0.036
	Sucrose, glucose,fructose metabolism	3-deoxyoctulosonate		0.21	-0.07	0.164	0.569	0.609	0.776
		erythrulose	C02045	**-0.76**	**-1.56**	0.019	0.294	0.000	0.002
		fructose	C00095	0.15	**-0.79**	0.392	0.721	0.000	0.005
		galactinol	C01235	0.06	-1.09	0.675	0.924	0.108	0.378
		galactitol (dulcitol)	C01697	0.40	0.30	0.278	0.662	0.345	0.622
		galactose	C01582	0.54	0.08	0.122	0.547	0.546	0.755
		mannitol	C00392	0.60	0.06	0.141	0.569	0.908	0.908
		mannose-6-phosphate	C00275	0.30	**-0.54**	0.191	0.594	0.011	0.090
		raffinose	C00492	**1.37**	**-1.56**	0.041	0.434	0.023	0.152
		sorbitol	C00794	0.56	0.00	0.344	0.690	0.952	0.915
		sucrose	C00089	**1.29**	**-1.69**	0.003	0.084	0.001	0.025

	C5 branched dibasic acid metabolism	citramalate	C00815	0.18	-0.79	0.318	0.688	0.000	0.006
Lipids	Free fatty acid	2-hydroxyglutarate	C02630	0.16	0.14	0.465	0.782	0.528	0.755
		2-hydroxypalmitate		-0.14	1.02	0.862	1.000	0.434	0.709
		2-hydroxystearate	C03045	-1.00	0.49	0.155	0.569	0.608	0.776
		3-hydroxypropanoate	C01013	0.25	0.83	0.751	0.951	0.064	0.314
		azelate (nonanedioate)	C08261	-0.51	-0.15	0.296	0.670	0.770	0.860
		cis-vaccenate (18:1n7)	C08367	0.14	0.08	0.319	0.688	0.463	0.710
		linoleate (18:2n6)	C01595	-0.92	0.70	0.277	0.662	0.603	0.776
		linolenate [alpha or gamma; (18:3n3 or 6)]	C06427	-1.00	1.04	0.326	0.690	0.443	0.710
		oleate (18:1n9)	C00712	0.10	-0.06	0.918	1.000	0.645	0.793
		undecanedioate		0.00	0.00	1.000	1.000	1.000	0.915
	Oxylipins	13-HODE + 9-HODE		-0.54	1.41	0.369	0.708	0.053	0.296
	Glycerolipids	1,2-propanediol	C00583	0.12	0.23	0.752	0.951	0.550	0.755
		1-palmitoylglycerol (1-monopalmitin)		0.24	0.01	0.342	0.690	0.973	0.915
		glycerol	C00116	0.26	1.34	0.602	0.886	0.011	0.090
	Phospholipids	1-linoleoylglycerophosphoinositol		-0.29	1.01	0.501	0.812	0.120	0.403
		1-palmitoylglycerophosphate	C04036	-1.12	0.75	0.290	0.670	0.453	0.710
		1-palmitoylglycerophosphocholine (16:0)		-0.92	0.59	0.295	0.670	0.741	0.847
		1-palmitoylglycerophosphoethanolamine		-0.86	0.94	0.368	0.708	0.491	0.738
		1-palmitoylglycerophosphoinositol		-0.71	0.96	0.320	0.688	0.452	0.710
		glycerol 3-phosphate (G3P)	C00093	0.12	0.01	0.504	0.812	0.992	0.915
		glycerophosphorylcholine (GPC)	C00670	0.12	0.18	0.759	0.951	0.650	0.795
		phosphoethanolamine	C00346	0.58	0.43	0.074	0.493	0.138	0.425
		2-linoleoylglycerophosphoinositol		-0.04	0.64	0.914	1.000	0.154	0.425
		1-palmitoylglycerophosphoglycerol		-0.81	0.92	0.267	0.662	0.289	0.567
		1-linolenoylglycerophosphoinositol		-0.69	0.93	0.148	0.569	0.339	0.622
		2-linolenoylglycerophosphoinositol		0.39	0.55	0.168	0.569	0.108	0.378
	Choline metabolism	choline phosphate	C00588	-0.20	0.36	0.506	0.812	0.130	0.425
		ethanolamine	C00189	0.01	-0.15	0.946	1.000	0.557	0.755
	Sphingolipid	phytosphingosine	C12144	0.00	0.53	1.000	1.000	0.320	0.622
	Sterols	beta-sitosterol	C01753	-0.04	-0.20	0.839	1.000	0.239	0.523
		campesterol	C01789	0.23	-0.14	0.420	0.753	0.528	0.755
		stigmasterol	C05442	-0.09	-0.01	0.680	0.924	0.880	0.894
Cofactors, Prosthetic Groups, Electron Carriers	CoA metabolism	pantothenate	C00864	0.00	0.70	0.924	1.000	0.010	0.090
	Nicotinate and nicotinamide metabolism	nicotinamide	C00153	-0.22	-0.10	0.358	0.707	0.705	0.831
		nicotinamide adenine dinucleotide (NAD+)	C00003	-0.23	-0.42	0.365	0.708	0.138	0.425
		nicotinamide riboside	C03150	-0.22	0.37	0.581	0.876	0.338	0.622
		nicotianamine	C05324	-0.04	0.24	0.654	0.910	0.540	0.755
		nicotinate ribonucleoside	C05841	0.18	0.19	0.282	0.662	0.258	0.532
		trigonelline (N'-methylnicotinate)	C01004	-0.09	0.36	0.614	0.888	0.061	0.308
	Oxidative phosphorylation	methylphosphate		0.03	0.33	0.999	1.000	0.412	0.698
		phosphate	C00009	-0.06	0.37	0.882	1.000	0.156	0.425
	Carnitine metabolism	carnitine	C00318	0.00	0.00	1.000	1.000	1.000	0.915
	Riboflavin and FAD metabolism	riboflavin (Vitamin B2)	C00255	0.58	0.77	0.259	0.662	0.148	0.425
		flavin mononucleotide (FMN)	C00061	0.34	0.33	0.258	0.662	0.206	0.481
	Ascorbate metabolism	5-ketogluconate	C01062	0.21	-0.51	0.651	0.910	0.232	0.519
		dehydroascorbate	C05422	0.96	0.40	0.009	0.183	0.257	0.532
		threonate	C01620	-0.30	-0.86	0.563	0.864	0.058	0.307
	Tocopherol metabolism	alpha-tocopherol	C02477	-0.07	-0.03	0.877	1.000	0.975	0.915
		beta-tocopherol	C14152	0.77	0.00	0.145	0.569	1.000	0.915
		delta-tocopherol	C14151	0.14	-0.25	0.850	1.000	0.540	0.755
		gamma-tocopherol	C02483	0.40	0.81	0.853	1.000	0.187	0.456
		gamma-tocotrienol	C14155	0.45	1.17	0.962	1.000	0.208	0.481
	Chlorophyll and heme metabolism	pheophorbide A	C18021	-0.12	0.52	0.836	1.000	0.599	0.776
		adenine	C00147	0.48	0.18	0.045	0.434	0.466	0.710
		adenosine	C00212	0.33	-1.25	0.546	0.844	0.025	0.157
		adenosine 5'-monophosphate (AMP)	C00020	0.08	-0.58	0.828	1.000	0.144	0.425

Nucleotide	Purine metabolism	N6-carbamoylthreonyladenosine		**0.38**	**0.92**	0.044	0.434	0.000	0.003
		allantoin	C02350	-0.12	**1.42**	0.866	1.000	0.066	0.314
		guanine	C00242	**1.99**	1.16	0.083	0.493	0.119	0.403
		guanosine	C00387	-0.03	0.01	0.841	1.000	0.951	0.915
		inosine	C00294	0.38	-0.04	0.186	0.588	0.887	0.894
		urate	C00366	**1.21**	0.72	0.079	0.493	0.243	0.526
		xanthosine	C01762	**0.51**	**0.77**	0.052	0.434	0.005	0.064
	Pyrimidine metabolism	cytidine	C00475	0.10	0.12	0.575	0.876	0.557	0.755
		pseudouridine	C02067	0.00	0.04	0.969	1.000	0.886	0.894
		uridine	C00299	-0.10	-0.04	0.459	0.782	0.699	0.831
		beta-alanine	C00099	**0.70**	-0.06	0.077	0.493	0.836	0.894
Peptide	gamma-glutamyl	gamma-glutamylglutamine	C05283	0.03	0.69	0.966	1.000	0.342	0.622
		gamma-glutamylisoleucine		0.07	**0.70**	0.782	0.972	0.011	0.090
		gamma-glutamylleucine		-0.04	0.50	0.700	0.934	0.262	0.532
		gamma-glutamylmethionine		0.00	0.00	1.000	1.000	1.000	0.915
		gamma-glutamylphenylalanine		-0.06	0.80	0.750	0.951	0.100	0.378
		gamma-glutamyltryptophan		0.10	0.66	0.984	1.000	0.262	0.532
		gamma-glutamylvaline		-0.15	**0.72**	0.545	0.844	0.005	0.064
Hormone metabolism	Abscisic acid metabolism	abscisate	C06082	**0.52**	0.40	0.097	0.493	0.187	0.456
	Gibberellin metabolism	gibberellate	C01699	**3.32**	0.00	0.000	0.000	1.000	0.915
	Ethylene metabolism	cyano-alanine	C02512	-0.43	0.33	0.162	0.569	0.522	0.755
Secondary metabolism	Alkaloids	loganin	C01433	-0.94	**-1.51**	0.132	0.568	0.008	0.080
	Benzenoids	2,4,6-trihydroxybenzoate		0.67	0.71	0.314	0.688	0.188	0.456
		benzyl alcohol	C00556	**1.13**	**-0.81**	0.033	0.390	0.048	0.278
		benzoyl-O-glucose		**2.03**	**-1.36**	0.003	0.084	0.047	0.278
		hydroquinone beta-D-glucopyranoside	C06186	0.04	-0.36	0.960	1.000	0.104	0.378
	Fatty acid and sugar derivatives	galactarate (mucic acid)		0.01	0.21	0.941	1.000	0.528	0.755
	Flavonoids	kaempferol 7-O-glucoside		0.00	0.00	1.000	1.000	1.000	0.915
		apigenin-7-o-glucoside	C04608	0.15	-0.22	0.761	0.951	0.621	0.783
		catechin	C06562	-0.54	**-1.60**	0.272	0.662	0.002	0.025
		epicatechin	C09727	**0.97**	0.26	0.062	0.462	0.390	0.667
		eriodictyol	C05631	**0.77**	0.14	0.062	0.462	0.728	0.847
		dihydroquercetin	C12316	-0.17	**-0.79**	0.637	0.902	0.054	0.296
		naringenin	C00509	0.14	0.10	0.699	0.934	0.813	0.894
		naringenin-7-O-glucoside		-0.23	**-0.49**	0.261	0.662	0.021	0.141
		procyanidin B1	C10238	-0.29	**-0.40**	0.200	0.615	0.077	0.344
		procyanidin B2	C17639	-0.34	-0.34	0.271	0.662	0.224	0.507
		quercetin	C00389	0.23	-0.34	0.683	0.924	0.536	0.755
		quercetin-3-galactoside	C10073	-0.42	0.06	0.212	0.643	0.910	0.908
		quercetin-3-o-glucoside	C05623	0.21	0.10	0.230	0.662	0.557	0.755
		rutin	C05625	0.24	**0.43**	0.118	0.541	0.010	0.090
		kaempferol-3-rhamnoside		1.03	-0.03	0.598	0.886	0.710	0.831
		catechin gallate		-0.47	**-2.32**	0.383	0.720	0.000	0.006
	Phenylpropanoids	ferulate	C01494	-0.04	**0.76**	0.984	1.000	0.089	0.378
		gallate	C01424	-0.47	**-1.74**	0.447	0.770	0.013	0.101
		resveratrol	C03582	-0.10	**-1.94**	0.956	1.000	0.000	0.006
		salicylate	C00805	0.00	0.28	0.951	1.000	0.366	0.635
		salidroside		-0.14	0.04	0.870	1.000	0.986	0.915
		salicin	C01451	0.41	0.20	0.312	0.688	0.880	0.894
		caffeate	C01197	-0.60	-0.62	0.465	0.782	0.354	0.632
	Tannins	procyanidin trimer		-0.14	-0.06	0.583	0.876	0.811	0.894
	Terpenoids	oleanolate		**-2.00**	**-1.56**	0.011	0.211	0.099	0.378
Xenobiotics	Chemical	trizma acetate	C07182	0.00	0.00	1.000	1.000	1.000	0.915
